# GNTI-122: an autologous antigen-specific engineered Treg cell therapy for type 1 diabetes

**DOI:** 10.1172/jci.insight.171844

**Published:** 2024-03-22

**Authors:** Gene I. Uenishi, Marko Repic, Jennifer Y. Yam, Ashley Landuyt, Priya Saikumar-Lakshmi, Tingxi Guo, Payam Zarin, Martina Sassone-Corsi, Adam Chicoine, Hunter Kellogg, Martina Hunt, Travis Drow, Ritika Tewari, Peter J. Cook, Soo Jung Yang, Karen Cerosaletti, Darius Schweinoch, Benjamin Guiastrennec, Eddie James, Chandra Patel, Tiffany F. Chen, Jane H. Buckner, David J. Rawlings, Thomas J. Wickham, Karen T. Mueller

**Affiliations:** 1 GentiBio Inc, Cambridge, Massachusetts, USA.; 2 Center for Immunity and Immunotherapies and the Program for Cell and Gene Therapy, Seattle Children’s Research Institute, Seattle, Washington, USA.; 3 Center for Translational Immunology, Benaroya Research Institute at Virginia Mason, Seattle, Washington, USA.; 4 IntiQuan GmbH, Basel, Switzerland.; 5 Department of Medicine,; 6 Department of Immunology, and; 7 Department of Pediatrics, University of Washington, Seattle, Washington, USA.

**Keywords:** Autoimmunity, Therapeutics, Diabetes, Gene therapy

## Abstract

Tregs have the potential to establish long-term immune tolerance in patients recently diagnosed with type 1 diabetes (T1D) by preserving β cell function. Adoptive transfer of autologous thymic Tregs, although safe, exhibited limited efficacy in previous T1D clinical trials, likely reflecting a lack of tissue specificity, limited IL-2 signaling support, and in vivo plasticity of Tregs. Here, we report a cell engineering strategy using bulk CD4^+^ T cells to generate a Treg cell therapy (GNTI-122) that stably expresses FOXP3, targets the pancreas and draining lymph nodes, and incorporates a chemically inducible signaling complex (CISC). GNTI-122 cells maintained an expression profile consistent with Treg phenotype and function. Activation of CISC using rapamycin mediated concentration-dependent STAT5 phosphorylation and, in concert with T cell receptor engagement, promoted cell proliferation. In response to the cognate antigen, GNTI-122 exhibited direct and bystander suppression of polyclonal, islet-specific effector T cells from patients with T1D. In an adoptive transfer mouse model of T1D, a mouse engineered-Treg analog of GNTI-122 trafficked to the pancreas, decreased the severity of insulitis, and prevented progression to diabetes. Taken together, these findings demonstrate in vitro and in vivo activity and support further development of GNTI-122 as a potential treatment for T1D.

## Abstract 2

GNTI-122 is a dual-engineered Treg therapy product developed for the treatment of type 1 diabetes with enhanced stability, tissue specificity, and selective IL-2 signaling.

## Graphical abstract

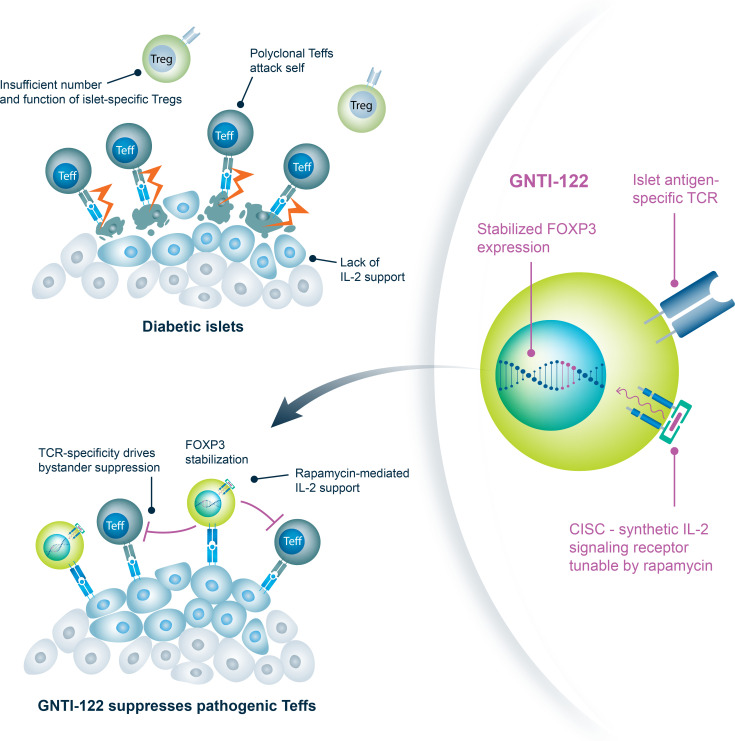

## Introduction

The past decade of drug development has been marked by exponential growth in cell therapies, with multiple regulatory approvals in oncology (1–4) establishing the foundation for clinical evaluation of new cell therapies for other disorders, including autoimmune and autoinflammatory diseases.

Type 1 diabetes (T1D) is a chronic autoimmune disease where T cell–mediated destruction of pancreatic islet insulin-producing β cells ultimately leads to insulin deficiency with symptomatic hyperglycemia. Poorly controlled hyperglycemia can result in irreversible multiorgan damage (5), as well as acute diabetic ketoacidosis and death. The pathogenesis of autoimmune diseases such as T1D is strongly associated with Treg dysfunction, driven in part by limited IL-2 availability and/or responsiveness (6–13).

Tregs play an essential role in maintaining peripheral immune tolerance through suppression of autoreactive immune effector cells. Polyclonal Tregs have been evaluated for the treatment of T1D in clinical studies in adults and children (14, 15). Importantly, these studies have shown that infusions of polyclonal Tregs were well tolerated, and the results suggested some signs of clinical benefit; however, efficacy was limited, leading to discontinuation in clinical development. The limited efficacy observed with polyclonal Tregs likely resulted from lack of pancreatic islet specificity and limited Treg proliferation, presumably attributable to local IL-2 scarcity (6–13), despite long-term detection of infused Tregs in the periphery.

In this study, we describe the development and testing of GNTI-122, an engineered Treg cell therapy. It is derived from autologous CD4^+^ T cells isolated from the patient’s peripheral blood, dual-engineered using homology-directed repair–based (HDR-based) genome editing to stably express FOXP3 and IGRP305-TCR, a T cell receptor (TCR) that recognizes the islet-specific glucose-6-phosphatase catalytic subunit–related protein (IGRP) peptide (16, 17), and includes a chemically inducible signaling complex (CISC) that provides IL-2 signaling support in response to rapamycin (18, 19). The resulting engineered Tregs (EngTregs) retain a stable regulatory phenotype, are activated in an islet antigen–specific manner, and can suppress islet-specific effector T cells (Teffs) in vitro. We further confirm long-term efficacy in a mouse model of diabetes, using mouse surrogate EngTregs (mEngTregs) with stable Foxp3 expression and an islet antigen–specific TCR. Importantly, the inclusion of CISC in the human EngTregs allows for production of dual-edited GNTI-122 cells with high purity and facilitates persistence of the adoptively transferred GNTI-122 cells in vivo (18, 19), providing cell-intrinsic IL-2 signaling designed to overcome limited IL-2 availability within the T1D pancreatic environment (6–13).

## Results

### Engineering of GNTI-122 via dual HDR–based genome editing of CD4^+^ T cells.

**Figure 1:**
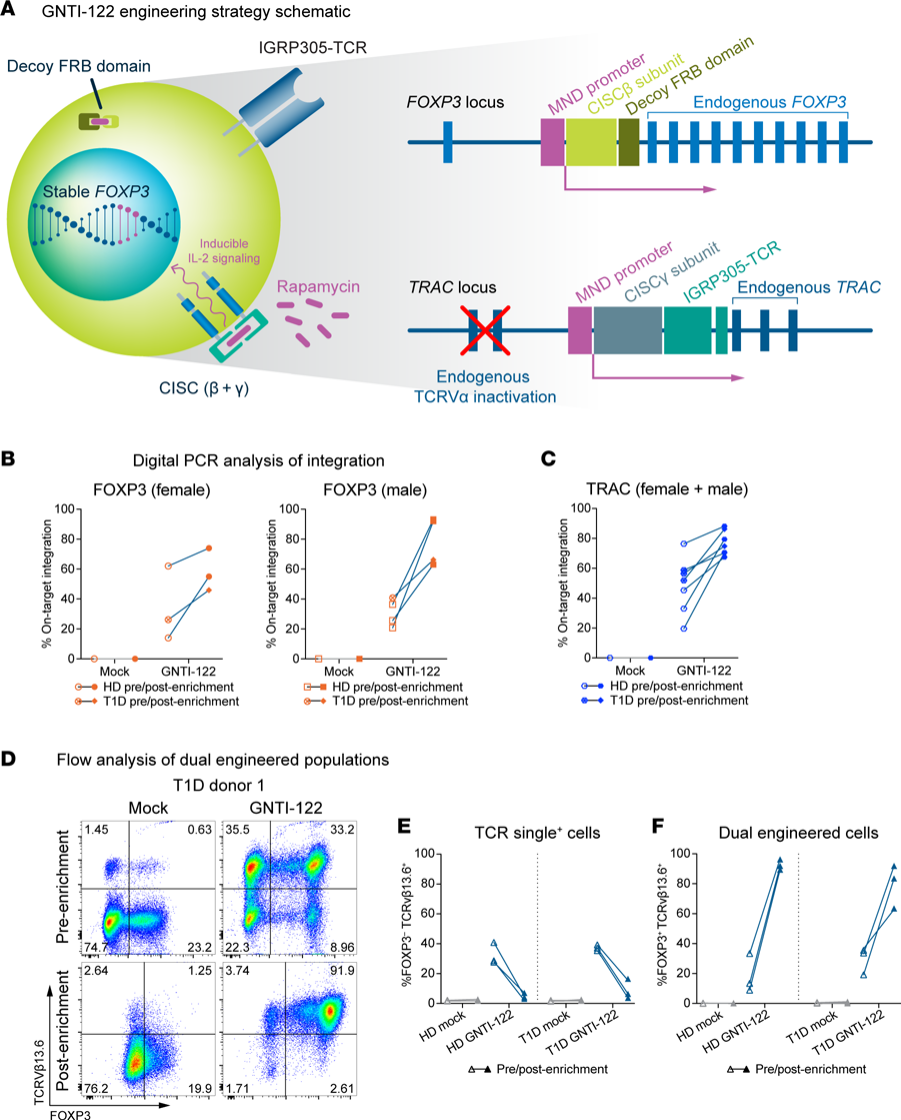
Genome engineering of GNTI-122 from CD4^+^ T cells. (**A**) Resulting genome-engineered product of GNTI-122. Digital PCR results of transgene integration at the (**B**) *FOXP3* and (**C**) *TRAC* loci of mock-engineered (Mock) and GNTI-122 cells from healthy donors (HDs) and patients with type 1 diabetes (T1D). (**D**) Representative FACS analysis of GNTI-122 and Mock cells generated from a patient with T1D, before enrichment to after enrichment with rapamycin (*n* = 3). Cells were stained 3 days after editing before enrichment to determine the initial TCRVb13.6^+^FOXP3^+^ editing frequency and at the time of cryopreservation after enrichment to determine purity. (**E** and **F**) Graphical representation of 3 independent batches of GNTI-122 cells and their corresponding Mock cells generated in parallel, from 3 HDs and 3 donors with T1D. Graphs represent the TCR single-positive population and dual-engineered populations. FRB, FKBP-rapamycin binding domain; MND, myeloproliferative sarcoma virus enhancer, negative control region deleted, dl587rev primer-binding site substituted; CISC, chemically inducible signaling complex.

GNTI-122 was engineered through dual HDR–based editing to stably express endogenous FOXP3 at the *FOXP3* locus and an islet antigen–specific TCR at the *TRAC* locus in bulk CD4^+^ cells from healthy donors and from donors with T1D. The IL-2 signaling CISC construct was split across both knockin transgenes to allow for selective enrichment of dual-engineered cells with rapamycin (Figure 1A and Supplemental Figure 1A; supplemental material available online with this article; https://doi.org/10.1172/jci.insight.171844DS1). GNTI-122 cells were characterized at the genomic level by digital PCR for integration of the MND-CISCβ-dFRB (previously described as the free FKBP-rapamycin-binding [FRB] domain) (20) at the *FOXP3* locus (Figure 1B) and the MND-CISCγ-IGRP305-TCR at the *TRAC* locus (Figure 1C). The initial on-target transgene integration frequencies for FOXP3 and TRAC were greater than that of mock-engineered (Mock) cells, and a measurable increase in percentage was observed after enrichment with rapamycin (Figure 1, B and C). Of note, the variability in FOXP3 editing efficiency can be attributed to male versus female donors and the presence of 1 versus 2 copies of the X chromosome, where *FOXP3* is located (Figure 1B). The values reflect an average of single and biallelic integrations of the total population for each locus.

While digital PCR allowed quantification of the frequency of engineered alleles at the population level, flow cytometry was implemented to measure both FOXP3 and IGRP305-TCR expression at single-cell resolution and to determine the frequency of dual-engineered cells. An antibody against TCRVβ13.6, the TCRβ V gene of the IGRP305-TCR, was used to measure editing at the *TRAC* locus. A ribonucleoprotein (RNP) complex–only control was also included in initial runs to show a loss in endogenous TCR expression upon editing (data not shown). The frequency of cells demonstrating expression of both FOXP3 and TCRVβ13.6 enriched via rapamycin-mediated CISC activation reached 92% for the final GNTI-122 cells (Figure 1, D–F, and Supplemental Figure 1B). The health of the cells after genome engineering was high, but decreased in viability as non–dual-engineered cells were selected out with rapamycin, as expected. The viability returned to greater than 90%, comparable to Mock, by the end of the process (data not shown). There were no significant differences in dual-engineering frequencies and enrichment efficiency between GNTI-122 generated from healthy donors or donors with T1D (donor information can be found in Table 1).

### Stable FOXP3 expression imparts Treg identity and function of the dual-engineered GNTI-122.

**Figure 2:**
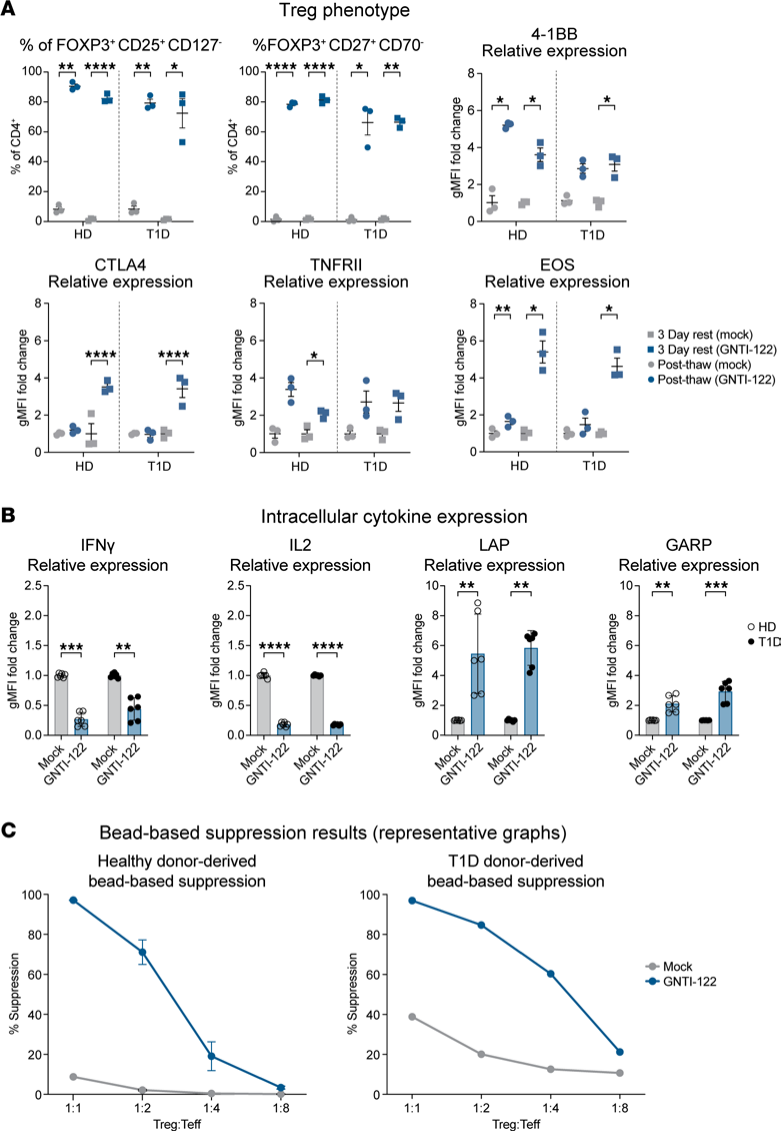
Stable FOXP3 expression imparts Treg phenotype and function. (**A**) Cells produced from 3 healthy donors (HDs) and 3 donors with type 1 diabetes (T1D) (total of 6 individual donors) were analyzed for flow cytometry immediately after thawing and after resting for 3 days. The percentages of each population (±SEM) are shown for GNTI-122 cells compared to mock-engineered (Mock) cells and are based on live CD4^+^ cells. The geometric MFI (gMFI) relative expression (mean ± SEM) for each marker is shown for the GNTI-122 cells compared to Mock cells. The MFI values for the Mock cells are based on CD4^+^, whereas the MFI values for the GNTI-122 cells are based on CD4^+^TCRVβ13.6^+^FOXP3^+^ (2-way ANOVA with Tukey’s multiple-comparison test). (**B**) Cells from 3 different HD and T1D donors were either stimulated with PMA/ionomycin/monensin for 5 hours and then stained (for intracellular IFN-γ and IL-2) or stimulated with anti-CD3/anti-CD28 activation beads for 24 hours and then stained (for surface LAP and GARP). Each cytokine was gated on either CD4^+^ for Mock cells or TCRVb13.6^+^FOXP3^+^ for GNTI-122 cells. Data presented as mean relative levels ± SD from duplicate measurements (2-way ANOVA with Šidák’s multiple-comparison test). (**C**) Representative bead-based suppression results from GNTI-122 cells generated from an individual HD and an individual donor with T1D (*n* = 3, mean ± SEM). *P < 0.05; **P < 0.01; ***P < 0.001; ****P < 0.0001. Teff, T effectors; PMA, phorbol 12-myristate 13-acetate; LAP, latency-associated protein; GARP, glycoprotein A repetitions predominant.

Insertion of the MND (myeloproliferative sarcoma virus enhancer, negative control region deleted, dl587rev primer-binding site substituted) promoter into the *FOXP3* gene bypasses endogenous regulatory elements, promotes the stable expression of *FOXP3*, and maintains an immunoregulatory Treg phenotype (Figure 2A and Supplemental Figure 2) (21). Compared with cultured Tregs (cTregs) expanded from FACS-isolated CD4^+^CD25^+^CD127^lo^ cells using previously established protocols (Supplemental Figure 3A), EngTregs utilizing this strategy have approximately 20-fold higher FOXP3 expression levels and no significant differences in the expression of key Treg markers such as CTLA-4, GITR (IKZF), TNFRSF18, and IL-2RA (Supplemental Figure 3B). Expression of HELIOS (IKZF2) trended lower, consistent with previous reports that thymic Tregs exclusively express this transcription factor (22–24). Importantly, this engineering strategy permits sustained high-level FOXP3 expression, with and without exposure to inflammatory conditions in vitro. In contrast, a significant proportion of cTregs cultured under identical conditions exhibited a reduction or loss of FOXP3 expression in the setting of inflammation and/or absence of IL-2 support (Supplemental Figure 3, C and D).

Consistent with EngTregs, GNTI-122 cells expressed high levels of FOXP3 and CD25 and low levels of CD127, a classic Treg signature (25). In addition, GNTI-122 maintained increased expression of TNFRII, which has been demonstrated to support stability of the Treg phenotype and promote Treg expansion by maintaining the cells in a functionally active state (26); GNTI-122 cells also have increased expression of CD27 and decreased expression of CD70, a phenotype aligned with a functionally stable natural Treg population (27). GNTI-122 maintained higher expression of EOS, 4-1BB, and CTLA-4 compared with Mock cells after 3 days of rest, indicative of functional Tregs (28, 29) (Supplemental Table 1).

Unlike Mock T cells, GNTI-122 expressed low levels of proinflammatory cytokines, including IL-2 and IFN-γ (Figure 2B). Stimulated GNTI-122 cells also showed significant upregulation of TGF-β–associated latency-associated peptide (LAP) and glycoprotein A repetitions predominant (GARP) compared with Mock cells, consistent with immunosuppressive function (Figure 2B). Importantly, the Treg identity and cytokine secretion profile were consistent between GNTI-122 cells produced from CD4^+^ cells obtained from a healthy control and from T1D donors (Figure 2, A and B). Further confirmation of Treg function was obtained via suppression of autologous polyclonal CD4^+^ Teff proliferation in response to CD3/CD28 stimulation, with findings consistent across both healthy control and T1D donors (Figure 2C).

### The expression of IGRP305-TCR imparts TCR specificity to GNTI-122, resulting in direct, bystander, and polyclonal suppression of islet-specific Teffs.

**Figure 3:**
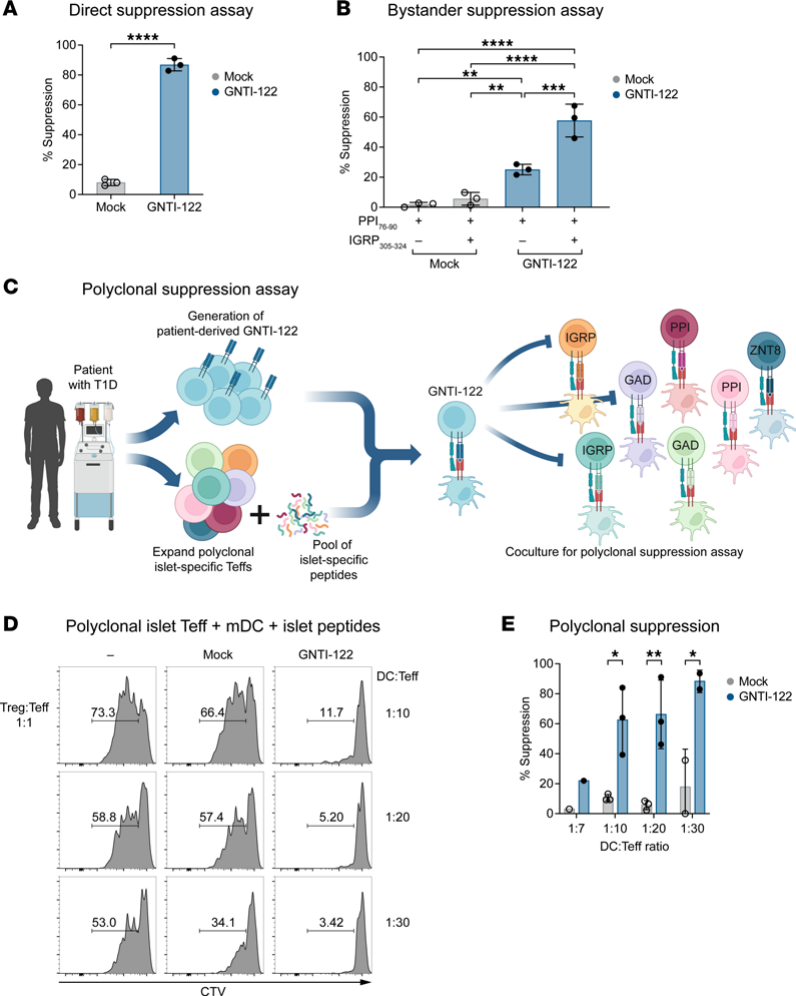
The expression of IGRP305-TCR imparts antigen-specific direct and bystander suppression of islet antigen–specific Teffs. Graphs include 3 individual donors of GNTI-122. GNTI-122 cells were cocultured with autologous Teffs from patient donors with T1D, and monocyte-derived dendritic cells as antigen-presenting cells (APCs). (**A**) Teffs express the same TCR, and APCs were loaded with their cognate peptide IGRP305-324 (for Tregs) (unpaired, 2-tailed *t* test). (**B**) Teffs express a different TCR, and the APCs were loaded with corresponding cognate peptide PPI_76–90_ (for Teffs) and IGRP_305–324_ (for Tregs) (2-way ANOVA). (**C**) Schematic diagram (created in BioRender.com) of the patient with T1D-derived pancreatic islet peptide–specific Teff polyclonal suppression assay. (**D**) Representative flow plots and (**E**) graphical representation of 3 independent experiments of polyclonal suppression assay where Teffs specific for 9 different cognate peptides were isolated, and APCs were loaded with their cognate peptides, including IGRP_305–324_, to activate GNTI-122 (unpaired, 2-tailed *t* test); mean ± SEM,*P < 0.05; **P < 0.01; ***P < 0.001; ****P < 0.0001. Mock, mock engineered; EngTregs, engineered T regulatory cells; mDC, monocyte-derived dendritic cells; CTV, CellTrace Violet.

Engineering of the IGRP305-TCR into GNTI-122 cells provides specificity for an islet β cell–derived self-antigen, the IGRP_305–324_ peptide presented by the MHC class II HLA-DRB1*04:01 allele (17). Target specificity of IGRP305-TCR was determined before selection for use in GNTI-122 (Supplemental Figure 4C) (17). When stimulated by its cognate peptide in a direct suppression assay, GNTI-122 significantly inhibited the proliferation of the IGRP305-TCR Teffs compared with Mock cells at a ratios of 1:1 T suppressors to T responders (Tsupp/Tresp) (Figure 3A and Supplemental Figure 4A). When cocultured with Teffs recognizing preproinsulin 76–90 (PPI_76–90_), a different β cell–specific antigen, GNTI-122, was able to significantly suppress the proliferation of the PPI76-TCR Teffs in the presence of the IGRP_305–324_ epitope (Figure 3B and Supplemental Figure 4B). Suppression was also observed with GNTI-122 when exposed to PPI_76–90_ in the absence of cognate peptide, although to a lesser extent (Figure 3B). These results show that GNTI-122 cells can directly suppress the proliferation of Teffs recognizing the same epitope presented by antigen-presenting cells (APCs), as well as the proliferation of Teffs recognizing a different islet epitope through bystander suppression. Most notably, GNTI-122 significantly inhibited the proliferation of autologous T1D–derived polyclonal Teffs specific to a pool of 10 different islet antigens in a polyclonal suppression assay (Figure 3, C–E). These results show that GNTI-122, with a single TCR specificity, can suppress the proliferation of pathogenic Teffs with TCR specificities that span a broad range of islet self-antigens and that target β cells and cause inflammation in patients with T1D. This in vitro assay provides a model for the mechanism of action in the local microenvironment of a patient’s pancreas or draining lymph nodes and supports the therapeutic potential of GNTI-122.

### Demonstration of TCR specificity and limited off-target cross-reactivity.

**Figure 4:**
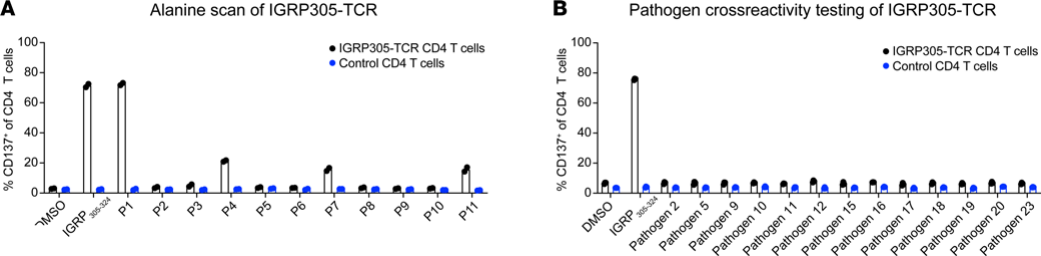
IGRP305-TCR is specific for its cognate peptide. K562/DR4 cells were pulsed with 1 μg/mL of the indicated alanine mutant peptides (**A**) or pathogen-derived peptides (**B**) and cocultured with IGRP305-TCR or control TCR-transduced T cells at a 3:1 effector/target ratio. CD137 expression was measured after 20 hours. The mean of 2 to 3 technical replicates is shown. Error bars denote 1 SD.

In addition to TCR on-target specificity, the potential cross-reactivity of IGRP305-TCR to other epitopes was also evaluated. First, the epitope residues critical for recognition by the IGRP305-TCR in the context of HLA-DRB1*04:01 were determined by assessing the response of IGRP305-TCR CD4^+^ T cells to the IGRP_305–324_ epitope and flanking residues, where each position in the minimal epitope 308–318 was individually mutated to alanine (Figure 4A and Supplemental Table 2). By measuring CD137 upregulation in response to artificial APCs pulsed with each of the mutants, P1-308 was identified as the only position that fully tolerated alanine substitution. Alanine substitution at all other positions either drastically reduced or eliminated the response of IGRP305-TCR CD4^+^ T cells, suggesting a high level of specificity of the therapeutic TCR.

Using the FLQIPTHEEH minimal epitope, an in silico screen was performed to define common human pathogen–derived peptides that had 3 or less amino acid differences from the IGRP_305_ minimal epitope (Supplemental Table 3). Each resulting peptide (*n* = 298) was then assessed for predicted binding to HLA-DRB1*04:01 using a previously published algorithm (17) and potential TCR contact residues within the minimal epitope. A panel of 13 pathogen-derived peptides across 11 species was assembled to assess potential cross-reactivity of the IGRP305-TCR (Supplemental Table 4). IGRP305-TCR CD4^+^ T cells were stimulated with the cognate and pathogen-derived peptides, similarly to the above assays. Consistent with the general lack of tolerated alanine substitutions, the IGRP305-TCR did not respond to any of the tested pathogen-derived epitopes (Figure 4B). Together, these data demonstrate the specificity of the IGRP305-TCR and mitigate safety concerns associated with suppression of anti-pathogen responses by GNTI-122.

### Murine T1D prevention with antigen-specific polyclonal suppression after adoptive transfer.

**Figure 5:**
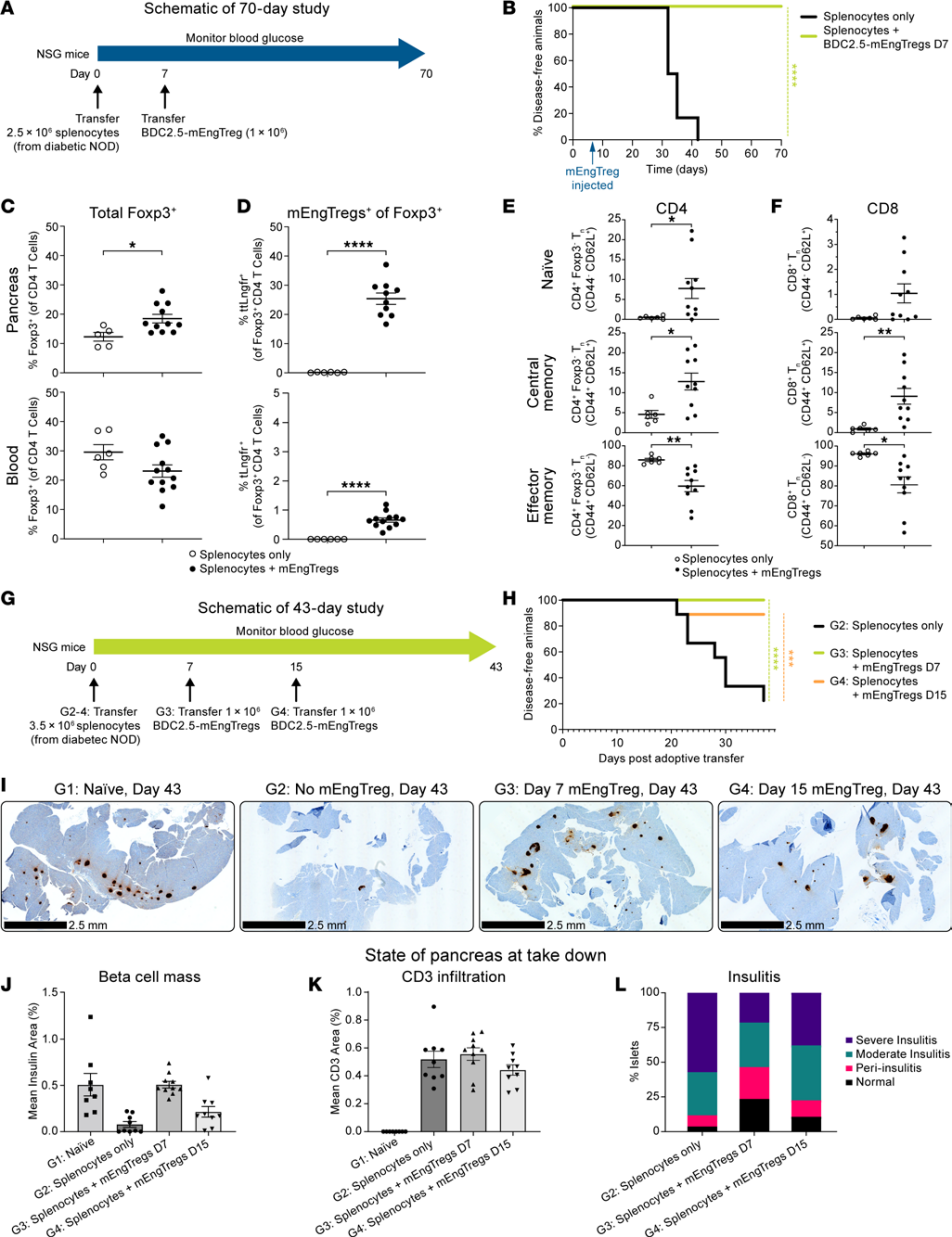
Antigen-specific murine engineered Tregs protect against an adoptive transfer model of diabetes, increase Treg levels in the pancreas, and reduce pancreatic effector memory T cells. (**A**) Experimental outline. (**B**) Kaplan-Meier curve displaying diabetes incidence in mice given only diabetogenic splenocytes (*n* = 6) or mice additionally given mTregs on day 7 (*n* = 12). Percentages of (**C**) Foxp3^+^ and (**D**) tLngfr^+^Foxp3^+^ cells in the pancreas and blood on day 70. Memory subsets within Foxp3^–^CD4^+^ T cells (**E**) or CD8^+^ T cells (**F**) in the pancreas on day 70. (**G**) Experimental outline. (**H**) Kaplan-Meier curve displaying diabetes incidence in mice given only diabetogenic splenocytes on day 0 (*n* = 10) and mice additionally given mEngTregs on day 7 (*n* = 10) or on day 15 (*n* = 10). (**I**) Representative immunohistochemistry for insulin. Scale bars: 2.5 mm. (**J**) β Cell mass of pancreata on day 43. (**K**) Quantification of CD3^+^ cell infiltration in pancreata on day 43. (**L**) Severity scores for islet inflammation (H&E staining). Approximately 20 islets quantified per mouse. Data are presented as mean ± SEM. Statistical analysis was by log-rank (Cox-Mantel) test; *P < 0.05; ***P < 0.001; ****P < 0.0001 by log-rank (Cox-Mantel) test (**B** and **H**) or unpaired, 2-tailed *t* test (**J** and **K**).

The concept of engineering Tregs with single-antigen specificity was evaluated in a mouse model of T1D (Figure 5, A and G) (17). To test this concept, we utilized the transgenic BDC2.5 nonobese diabetic (NOD) mouse, which expresses the BDC2.5 TCR on CD4^+^ T cells specific for 2.5 hybrid insulin peptide antigen (30). BDC2.5 mEngTregs were prepared from BDC2.5 CD4^+^ cells gene engineered with an MND promoter to drive the expression of truncated Lngfr (tLngfr) and endogenous Foxp3 (Supplemental Figure 5A), but without the CISC activation support due to the lack of a functional murine version. Using a polyclonal diabetogenic splenocyte adoptive transfer model into NOD-*scid* Il2Rgamma^null^ (NSG) mice, a single dose of 1 × 10^6^ BDC2.5-mEngTregs on day 7 was able to prevent the development of hyperglycemia in 100% of the mice through day 70. In contrast, 100% of the mice in the control group developed hyperglycemia by day 42 after splenocyte transfer (Figure 5B). On day 70, the BDC2.5-mEngTregs were present in the pancreas (Figure 5, C and D), indicating trafficking and persistence. Although CD4^+^ and CD8^+^ T cells were present in the pancreas on day 70 in mice that received BDC2.5-mEngTregs (Figure 5, E and F), these mice retained glucose control (Figure 5B). These combined findings support the conclusion that BDC2.5-mEngTregs manifest a sustained tolerogenic effect on pathogenic Teffs in the local pancreatic microenvironment.

In a separate study, we compared mEngTreg dosing on day 7 versus day 15. First, the kinetics of insulitis (Supplemental Figure 5B) and reduction in islet β cell mass mediated by T1D splenocyte transfer were assessed at 7 and 15 days after adoptive transfer in the absence of mEngTregs. The percentage of islets with insulitis was greater on day 15 than on day 7, the former with only 18.1% of islets scoring normal (Supplemental Figure 5, C and D). We next assessed the impact of 1 × 10^6^ BDC2.5-mEngTregs delivered on day 7 or 15 after splenocyte transfer (Figure 5G). Animals treated with BDC2.5-mEngTregs on day 7 or day 15 exhibited protection from hyperglycemia (100% and 89%, respectively; Figure 5H) up to day 43, when the surviving cohorts were sacrificed for analysis. In contrast, only 20% of the untreated mice survived until the endpoint. Both treatment groups exhibited reduced severity of insulitis and retention of islet β cell mass compared with untreated mice. Protection was most evident in mice treated with BDC2.5-mEngTregs on day 7 (Figure 5, I–L, and Supplemental Figure 5E).

### In vitro and in vivo cell kinetics of GNTI-122.

**Figure 6:**
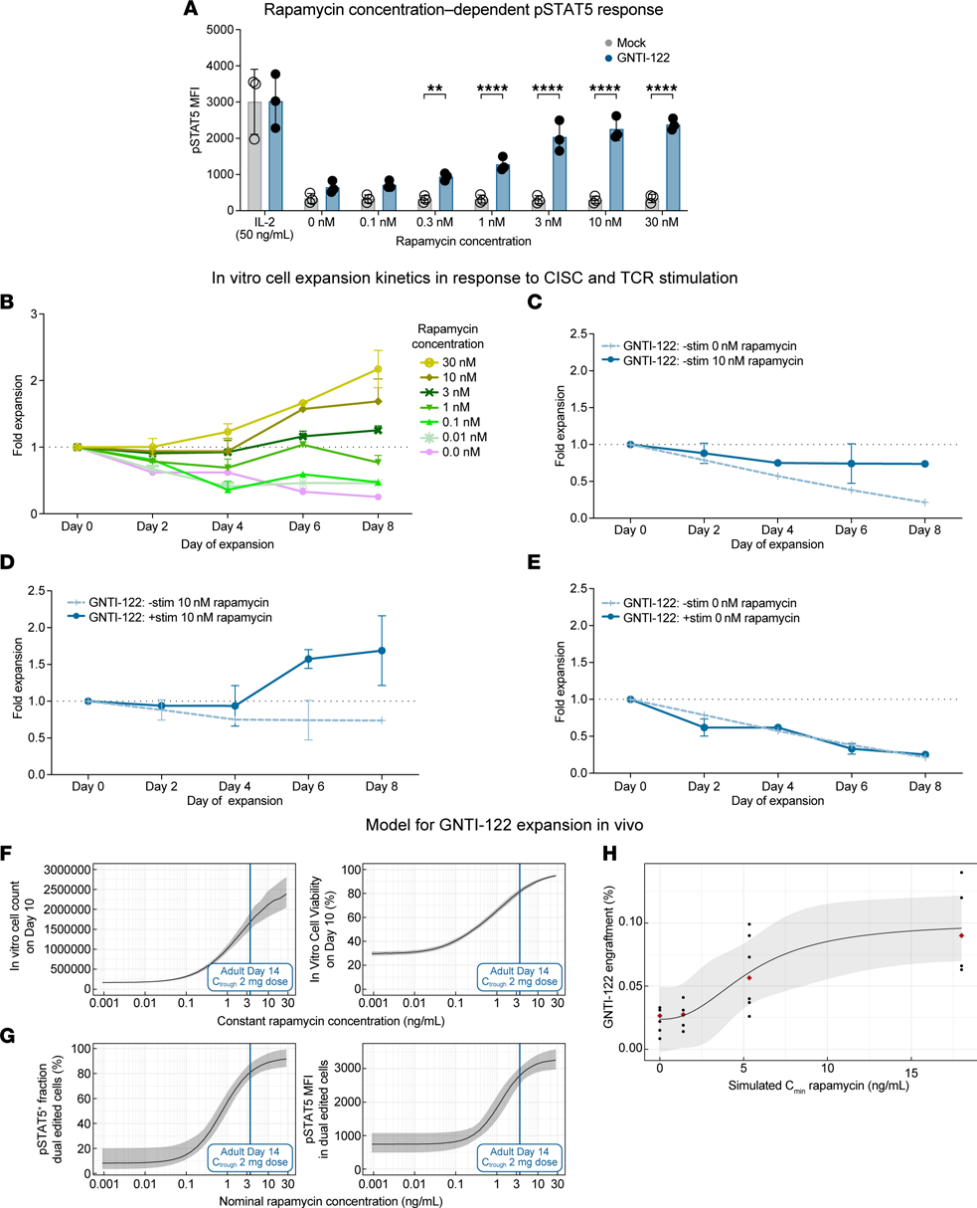
CISC activation enables rapamycin-mediated IL-2–like signaling to control GNTI-122 proliferation and viability in the absence of IL-2. (**A**) Rapamycin concentration–dependent phosphorylation of STAT5 by CISC activation. MFI of p-STAT5 was quantified at each concentration of rapamycin. Repeated-measures ANOVA with Šidák’s multiple-comparison tests at each dose. **P < 0.01, ****P < 0.0001. Data are presented as mean ± SEM (*n* = 3 donors). (**B**–**E**) GNTI-122 cell expansion in vitro was measured over 10 days; data presented as mean ± SEM (*n* = 2) from representative donors. (**B**) Chemically inducible signaling complex (CISC) activation by 10 nM rapamycin improved cell survival in the absence of TCR signaling. (**C**) Both CISC activation (10 nM rapamycin) and TCR signaling were required for cell proliferation. (**D**) TCR stimulation alone, in the absence of CISC activation by 10 nM rapamycin, did not support cell survival. (**E**) Rapamycin exposure–dependent proliferation of GNTI-122 in the presence of TCR stimulation. Simulation of rapamycin concentration-response relationship of in vitro GNTI-122 cell (**F**) expansion and viability, and (**G**) CISC activation (p-STAT5 percentage positive cells and MFI) alongside predicted rapamycin trough exposures (C_min_) in adult subjects at the 2-mg rapamycin daily dose. Median and 90% CI were derived from 500 population mean parameters sampled from the uncertainty distribution of the parameter estimates. (**H**) Model of rapamycin exposure-response for GNTI-122 engraftment in NSG mice on day 19, shown as a function of rapamycin trough (C_min_) concentrations. Black dots indicate observed GNTI-122 engraftment. Red diamonds correspond to median engraftment per simulated rapamycin exposure. Black line and ribbon indicate the model fit and 68% prediction interval.

CISC activation supports efficient GNTI-122 enrichment and expansion by providing an IL-2 signal to only dual HDR–edited EngTregs (Figure 1C). CISC activation induces phosphorylation of STAT5 in a rapamycin concentration–dependent manner (Figure 6A). Importantly, Mock and GNTI-122 cells exhibited similar responses to IL-2, indicating that this engineering strategy had no detrimental impact on the ability of GNTI-122 to respond to natural IL-2, as determined by similar levels of p-STAT5 (Figure 6A). Similar to the p-STAT5 results, GNTI-122 expansion kinetics over 8 days were also dependent on rapamycin concentration (Figure 6B). Similar to IL-2 signaling in T cells, CISC activation–mediated IL-2 signaling supports the in vitro persistence and proliferation of GNTI-122 only in the presence of TCR stimulation (Figure 6, C–E).

To directly assess the potential impact of the CISC in vivo, we evaluated whether CISC activation promoted engraftment and persistence of GNTI-122 using an NSG mouse model in the setting of rapamycin treatment (Supplemental Figure 6A). Note that although rapamycin was predicted to support IL-2–like signaling and cell survival in the absence of exogenous IL-2, the lack of TCR engagement (in the absence of human MHC class II and cognate antigen) was anticipated to improve short-term engraftment but not lead to EngTreg expansion. As predicted, rapamycin led to a dose-dependent increase in GNTI-122 engraftment (Supplemental Figure 6B), signifying the ability for rapamycin to induce an IL-2–like signal through CISC activation in vivo to support engraftment.

### Mathematical modeling to characterize in vitro cell kinetics and exposure-response and to predict in vivo GNTI-122 expansion in response to rapamycin.

In clinical studies, rapamycin could be used for a short duration to deliver IL-2–like signaling through CISC activation in GNTI-122 to compensate for the IL-2 scarcity in autoimmune conditions. We therefore utilized the data obtained from the in vitro and in vivo GNTI-122 kinetics studies to mathematically model the clinical rapamycin dosing anticipated to be required to support engraftment of GNTI-122 in patients with T1D. First, a nonlinear mixed effects model was developed that could describe expansion and viability of GNTI-122 cells under various in vitro culture conditions encompassing various combinations of rapamycin exposures and TCR stimulation. The model showed that GNTI-122 cells can expand in a rapamycin-dependent manner in the presence of TCR stimulation. Neither rapamycin nor TCR stimulation alone could trigger GNTI-122 proliferation, indicating that both signals are required for the cells to expand. The model further predicted that rapamycin reduced the rate of cell death of GNTI-122 independently of TCR stimulation and in the absence of exogenously added IL-2, which is critically required by Tregs for survival. Modeling simulations exemplifying the GNTI-122 cell growth and viability on day 10 as a function of rapamycin concentration are provided in Figure 6F. The activation of CISC receptor, quantified via fraction of cells with active STAT5 signaling and the p-STAT5 mean fluorescence intensity (MFI) were both described by sigmoidal E_max_ models with baseline, which show that rapamycin concentrations to achieve half-maximum CISC activation were estimated to be 0.80 ng/mL and 1.25 ng/mL, respectively (Figure 6G).

Second, we developed and qualified an in vitro CISC activation exposure-response model and an in vivo GNTI-122 engraftment model in mice based on internal data. Overall, an increase in GNTI-122 cell growth and viability was predicted for rapamycin levels as low as 0.1 ng/mL, and the onset of saturation was predicted to take place at approximately 10 ng/mL (Figure 6F). The response in CISC activation marker p-STAT5 showed a similar trend, with a predicted onset of STAT5 activation around 0.1 ng/mL and a predicted onset of saturation for rapamycin concentrations around 3 to 4 ng/mL (Figure 6G). In vivo, GNTI-122 engraftment on day 19 in mouse blood was modeled with a sigmoidal E_max_, for which the half-maximum engraftment was estimated at 5.5 ng/mL (Figure 6H), which was in the range of the simulated rapamycin trough concentration for the 0.05 mg/kg dose group (Figure 6C). Overall, in vivo engraftment was found to be 3.2-fold greater with rapamycin on a once every other day schedule compared with no rapamycin.

### Translational model-based approach to guide clinical dosing of rapamycin in combination with GNTI-122 in patients with T1D.

**Figure 7:**
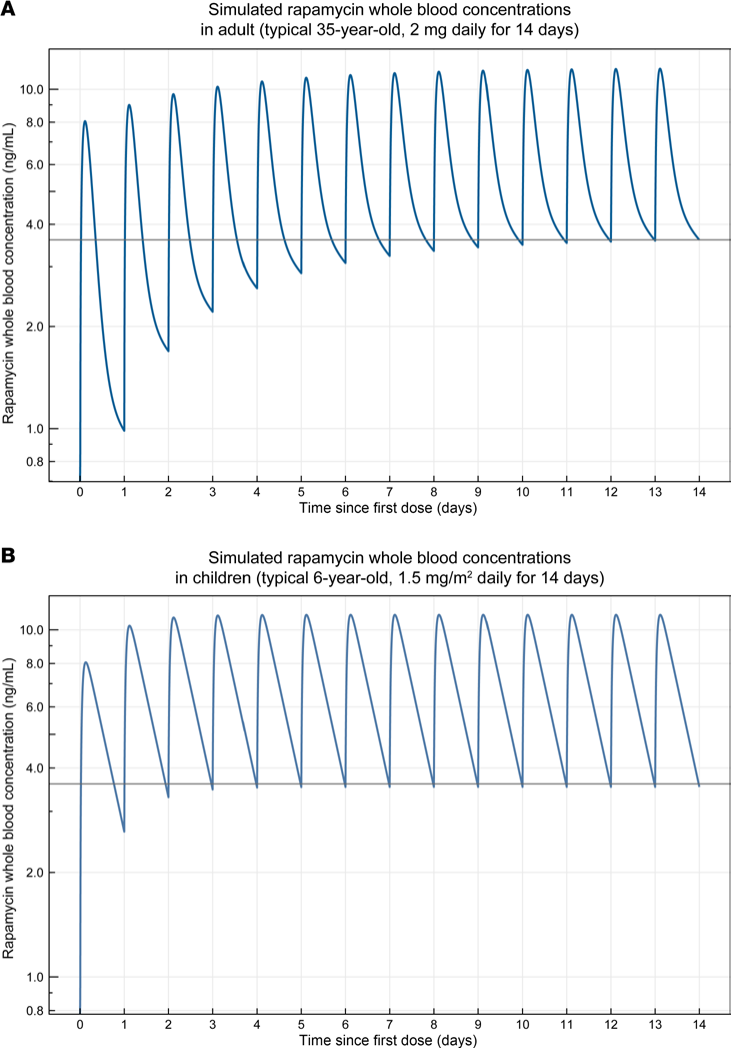
Simulation of rapamycin dosing in adult and pediatric patients to achieve rapamycin exposures predicted to support GNTI-122 engraftment. Simulated whole-blood rapamycin concentrations representing rapamycin exposures over the course of 14-day daily treatment regimen (**A**) in a 35-year-old adult at 2 mg per day dose and (**B**) in a 6-year-old child at 1.5 mg/m^2^ per day dose. The solid line depicts the 3.6 ng/mL trough concentration predicted to promote GNTI-122 engraftment in patients.

Published rapamycin pharmacokinetics (PK) models in humans were evaluated and qualified based on an external set of published PK data. As a result of the literature search, 2 separately published rapamycin PK models were qualified and selected, one for adults (31) and one for children and adolescents (32). The published adult model was extended to include a tablet/solution formulation effect on the absorption rate constant, as previously published (33), to account for the intended clinical formulation. A human target rapamycin trough exposure range of 3 to 4 ng/mL was selected with the idea to support engraftment while leaving some margin for potential dose escalation. Based on the model simulations, this target could be achieved with a daily rapamycin dose of 1.5 mg/m^2^ body surface area (BSA) in children or a daily dose of 2 mg for adolescents and adults. An illustration of the simulated typical rapamycin blood PK profiles for each age group is presented by dose in Figure 7. In addition, the predicted effect of age on the typical trough concentrations in children and adolescents is presented in Supplemental Figure 7. Using this approach, a target rapamycin trough exposure of 3 to 4 ng/mL in humans was anticipated to be necessary to support cell engraftment in patients. To achieve the target rapamycin trough exposure, a dose of 2 mg daily was selected for adolescents (≥13 to <18 years of age) and adults (≥18 years of age), and a dose of 1.5 mg/m^2^ BSA was selected for children (≥3 to <13 years of age) using the selected human PK models. Importantly, the doses of rapamycin proposed here are below therapeutic trough concentrations (5–15 ng/mL) measured for rapamycin in approved indications (34) and are projected to be used for short, periodic durations (~2 weeks).

## Discussion

Evidence from both preclinical (17, 21) and clinical (14) studies have shown the potential for using Tregs to treat autoimmune or autoinflammatory disorders such as T1D. However, previous and current clinical trials in T1D utilize Treg products that lack the target recognition critical for Tregs to function at intended tissue sites and do not provide IL-2 support within an IL-2–deficient pancreatic environment. Thymic Tregs also face the challenge of maintaining FOXP3 expression required for a sustained Treg phenotype (14, 15, 35). Here, we build upon previously described methods of engineering Tregs (16, 17, 19–21) and demonstrate successful engineering of bulk CD4^+^ T cells from patients with T1D to generate a cell product, GNTI-122, that overcomes these major challenges. The data presented here provide a nonclinical data set supporting its application in combination with rapamycin to treat patients with recently diagnosed T1D in the clinic.

The ability to stabilize FOXP3 expression and maintain the immunoregulatory phenotype from bulk CD4^+^ cells is critical for successful therapies. To date, thymic Treg adoptive transfer therapies have not demonstrated persistence required for efficacy (14, 15), possibly due to an unstable regulatory phenotype in vivo and impaired IL-2 responsiveness (35). The engineering approach utilized to generate GNTI-122 functions independently of Treg-specific demethylated region (TSDR) methylation status. This key feature protects engineered cells from FOXP3 silencing and maintains FOXP3 expression for stable regulatory function, as demonstrated by the Treg phenotype and function of the engineered cells. A surrogate of EngTregs was developed using NOD-BDC2.5 CD4^+^ cells for proof of principle of antigen-specific murine engineered Tregs by knocking in the same MND promoter to express FOXP3 similarly to GNTI-122, albeit without CISC. In vivo, adoptive transfer of mouse surrogate EngTregs allowed for Treg persistence up to day 70 without CISC activation, supporting the stability of the FOXP3 engineering at the dose used in this model. Overall, these combined data provide evidence that engineered Tregs with single islet antigen specificity can traffic to and exert immunosuppressive effects on the pancreas and reduce β cell destruction caused by pathogenic Teffs. These findings further underscore the predicted long-term stability of the Treg phenotype and function in EngTregs with potential for maintaining long-term tolerance.

Although polyclonal Tregs are known to be safe and well tolerated, the lack of tissue specificity limits effective disease treatment. By engineering the IGRP305-TCR directly into the TCRα constant domain, specificity for the target organs is imparted while reducing mispairing with endogenous TCRα chains. As demonstrated previously, different TCRs drive different regulatory functions, as measured by bystander and polyclonal suppression assays, demonstrating antigen-specific activation and suppression of Teffs specific to other pancreatic islet–specific antigens (17). Interestingly, GNTI-122 did show significant bystander suppression in the absence of its cognate peptide. This is consistent with the current understanding of the mechanism of suppression, as GNTI-122 still expresses high levels of CD25, LAP, and GARP, having been exposed to TCR stimulation during the production process. The polyclonal suppression assay utilized here enabled selection of top-performing TCRs with the desired T1D therapeutic activity, as exemplified by IGRP305-TCR in GNTI-122.

The IGRP305-TCR recognizes the target epitope (IGRP_305–324_) when presented by class II MHC (HLA-DRB1*04:01 allele). As most somatic cells do not express MHC class II without inflammation and lack expression of IGRP, the potential for off-target activation of the IGRP305-TCR is likely absent or minimal, supporting the safety profile for GNTI-122. The IGRP protein is specifically expressed by β cells and, to a lower extent, by α cells in the pancreatic islet. In ongoing pancreatic islet inflammation characteristic of patients with T1D, IGRP released from damaged β islet cells can be processed by professional APCs at draining lymph nodes and presented to GNTI-122. DR4 is present in approximately 60% of patients with T1D, and the specific HLA-DRB1*04:01 allele is present in a significant population of patients recently diagnosed with T1D. Thus, GNTI-122 can be used as a therapeutic agent in a large proportion of T1D patients.

IL-2 scarcity and IL-2 signaling defects are hallmarks of T1D pathology shown both clinically (35–40) and in preclinical settings (41). These deficits impair Treg homeostasis and survival, leading to reduced function and an imbalance in the Treg/Teff ratio. Restoring IL-2 support can reestablish Treg numbers, function, and survival, leading to amelioration of T1D in preclinical models (41). It is important to note that because the mEngTreg used in the in vivo studies did not contain IL-2 signaling support via CISC, the resultant activity likely represents an underestimate of the activity attainable in humans. We believe the cell engineering strategy described here is unique because the inclusion of CISC activation allows delivery of IL-2–like signaling support specifically to the GNTI-122 cells. Rapamycin-mediated CISC activation provides 2 major features; it allows for enrichment of a highly pure, dual-engineered GNTI-122 therapeutic product and also supports GNTI-122 activation, engraftment, and potential for long-term persistence in vivo. Importantly, inclusion of CISC activation also enables restoration of IL-2 signaling specifically to GNTI-122 while avoiding expansion of endogenous Tregs or Teff and NK cells that could be associated with broad immunosuppression or exacerbation of disease, respectively. Furthermore, GNTI-122 is predicted to expand and become activated through combined IL-2 and TCR signaling, which is anticipated to occur only at the sites of disease (pancreas and pancreatic lymph nodes), providing a highly specific therapy with reduced off-tissue effects.

Recent data highlight the significance of immune intervention that is initiated prior to T1D clinical diagnosis (42), but despite years of research to identify metabolic, genetic, and environmental factors that may be amenable to intervention, there is currently no cure for T1D. While the onset of T1D can occur at any age, diagnosis in pediatric patients occurs in 2 peaks between 4 and 6 years and 9 and 12 years, with a mean age of 8 years (43, 44). Pediatric patients often present with hallmark symptoms of uncontrolled hyperglycemia, including polyuria and polydipsia, with approximately one-third presenting with diabetic ketoacidosis, a life-threatening conditioning associated with poorer outcomes (5, 45). Importantly, the clinical presentation in pediatric patients reflects the more aggressive autoimmune pathology in this age group; β cell destruction occurs more rapidly in younger patients, with faster disease progression compared with adults (46, 47). To model aggressive disease progression marked by pathogenic Teff infiltration within the pancreas, we utilized an adoptive transfer mouse model using diabetic Teffs triggering rapid and severe insulitis. In this stringent setting, antigen-specific EngTregs were able to prevent disease progression. Importantly, recent data highlight the significance of immune intervention initiated before clinical T1D diagnosis (42), an approach that could be implemented with a Treg therapy.

Together, our findings provide a nonclinical data set that supports the potential clinical use of GNTI-122 in combination with rapamycin in patients with recently diagnosed T1D. Our in vitro findings showing suppression of T1D Teffs with broad islet specificity and in vivo studies showing protection in T1D animal models support the concept that adoptive therapy using GNTI-122 may be sufficient to reestablish the Teff/Treg balance and preserve β cell function. This combined work demonstrates that GNTI-122 effectively overcomes the challenges associated with current Treg therapeutics and may hold significant clinical potential in treatment of T1D, including addressing the significant unmet clinical need in pediatric patients.

## Methods

### Sex as a biological variant.

In vivo mouse surrogate studies using diabetogenic splenocytes were conducted with female NOD mice due to their increased propensity for disease onset. To minimize alloreactivity between female donors in male recipients, female NSG recipients were used with female BDC2.5-mEngTregs. GNTI-122 engraftment was assessed in female NSG mice for animal welfare since female mice better tolerate the irradiation. No other sex-specific factors influencing the results are expected. Both male and female human healthy donors and patients of T1D were utilized to generate GNTI-122 to show no differences in engineering feasibility, phenotype, and function of the resulting cells. When measuring on-target integration using digital PCR, donor sex was considered when reporting integration frequency to account for the *FOXP3* gene located on the X chromosome.

### Cell engineering.

The GNTI-122 engineering strategy was adopted from methods developed in the Rawlings laboratory (20). CD4^+^ T cells were isolated from 3 healthy donors and 3 donors with T1D by negative selection using the EasySep human CD4^+^ T-Cell Isolation Kit (STEMCELL Technologies) and cryopreserved in CryoStor 10 (STEMCELL Technologies). On day 0, the CD4^+^ T cells were thawed and activated using Dynabeads Human T-Expander CD3/CD28 (Thermo Fisher Scientific) per manufacturer’s recommendations. Following activation, cells were gene edited by electroporating with 2 RNPs; each RNP consisted of Cas9 protein (Aldevron) and an sgRNA (BioSpring) targeting either the *FOXP3* or *TRAC* locus; transgene templates carrying MND-CISCβ-dFRB for *FOXP3* loci and MND-CISCγ-IGRP305-TCR for the *TRAC* loci were delivered via adeno-associated virus serotype 6 (AAV6) (Supplemental Figure 1A). Successfully dual-engineered cells were enriched and expanded with rapamycin and a secondary activation step, and cryopreserved after purity exceeded 80%. GNTI-122 cells and their corresponding Mock cells, which were electroporated but not treated with RNP or AAV6, were analyzed by flow cytometry during the production process 3 days after editing and on the day of cryopreservation to determine the editing efficiency and purity.

### Digital PCR.

Integration frequency of MND-CISCβ-dFRB at the *FOXP3* locus and the MND-CISCγ-IGRP305-TCR at the *TRAC* locus were determined by digital PCR. The on-target integrations were normalized to a single-copy reference gene, either *GAPDH* or ribonuclease P RNA component H1 (*RPPH1*). Genomic DNA from 1 million cells was collected using the DNeasy Kit (Qiagen) on day 7 and the day of cryopreservation to evaluate pre- and postenrichment time points. Healthy donor samples were run on the QX-200 (Bio-Rad) and analyzed using the included QuantaSoft software. T1D patient donor samples were run on the Absolute Q (Thermo Fisher Scientific) and analyzed on its software. Primer and probe sequences can be found in Supplemental Tables 5 and 6.

### Flow cytometry.

Dual engineering and the Treg phenotype were evaluated by flow cytometry. Samples for flow cytometry were all processed for intracellular staining using the eBioscience FOXP3 Transcription Factor Staining Buffer Set and conducted per the manufacturer’s protocol. For phenotyping, cryopreserved GNTI-122 cells and their corresponding Mock cells were thawed and stained, and flow cytometry was performed immediately after thawing and after a 3-day rest in low–IL-2 medium (PeproTech, 5 ng/mL). Markers of Treg function, activation, exhaustion, and stability were investigated (detailed in Supplemental Table 1). Specific antibody information can be found in Supplemental Tables 7 and 8.

### Cytokine profile assessment of GNTI-122 cells.

Cryopreserved GNTI-122 cells and their corresponding Mock cells were thawed and rested in IL-2 (PeproTech, 5 ng/mL) for 2 days. Cells were washed to remove residual IL-2 and rested for an additional 24 hours. Cells were stimulated with either anti-CD3/anti-CD28 Dynabeads (Gibco) at a bead-to-cell ratio of 2:1 for 24 hours at 37°C, or 20 ng/mL PMA (Sigma-Aldrich), 1 μg/mL ionomycin (Sigma-Aldrich), and 1 μg/mL monensin (Invitrogen) for 5 hours at 37°C. Cells treated with anti-CD3/anti-CD28 beads were stained with anti-LAP (BioLegend) and anti-GARP (BD Biosciences) antibodies, and cells stimulated with PMA, ionomycin, and monensin were stained with anti–IFN-γ and anti–IL-2 (BioLegend) antibodies for flow cytometry as described above.

### Bead-based, direct, bystander, and polyclonal suppression assays.

Bead-based (antigen-agnostic) suppression assays (21) and direct, bystander, and polyclonal (antigen-specific) suppression assays (17) were conducted as previously reported. Suppression percentages were calculated as follows: suppresion (%) = ([*a* – *b*]/*a*) × 100, where *a* is the percentage Teff proliferation in the absence of Tregs, and *b* is the percentage of Teff proliferation in the presence of Tregs.

### TCR cross-reactivity qualification assays.

TCR selection (see Supplemental Methods) and qualification were performed using methods previously published (17). Specificity of the IGRP305-TCR recognizing IGRP_305–324_ was investigated by expressing it on primary human CD4^+^ T cells and testing for reactivity against alanine mutants of the cognate epitope and similar amino acid motifs present in pathogen-associated proteins.

### In vitro STAT5 signaling and CISC activation studies.

GNTI-122 and Mock cells were thawed and rested for 4 days in the presence of IL-2 (5 ng/mL) then cytokine starved for 3 days. The cells were then exposed to rapamycin at concentrations from 0 to 30 nM for 1 hour. Cells exposed to IL-2 alone served as the positive control. STAT5 phosphorylation, a marker of IL-2 pathway activation, was determined after fixation and permeabilization with cold methanol by intracellular staining for p-STAT5 using the BD CytoFix/Cytoperm and Perm Buffer III kits via flow cytometry.

### Adoptive transfer T1D mouse model.

In the adoptive transfer T1D mouse model, 2.5 million splenocytes from diabetic female NOD mice (NOD/ShiLtj, Jackson Laboratory) were transferred into 8-week-old female NSG mice (Jackson Laboratory) (day 0), as previously described (17). Each mouse received vehicle only or 1 million female BDC2.5-EngTregs (GNTI-122 mouse surrogate) intravenously in 0.2 mL PBS (Figure 5A) on day 7. Blood glucose was measured twice per week and body weight once per week. Mice were scored as diabetic after 2 consecutive readings of 250 mg/dL or greater and euthanized when blood glucose levels exceeded 500 mg/dL. Blood and pancreas were harvested, processed, and evaluated by flow cytometry as described above. Pancreata were also fixed in 10% neutral buffered formalin and processed for histological analysis of islet inflammation (insulitis) and β cell mass. Insulitis was scored on H&E-stained sections per the scoring system described in Morimoto et al. (48). Briefly, at least 15 islets (or as many as were present) were analyzed) across 4 different sections per mouse. Insulitis was scored as follows: 0, normal; 1, peri-insulitis (mononuclear cells surrounding islets and ducts, but no infiltration of the islet architecture); 2, moderate insulitis (mononuclear cells infiltrating <50% of the islet architecture); and 3, severe insulitis (mononuclear cells infiltrating >50% of the islet architecture). Islet β islets and T cells were visualized by antibody staining for insulin and CD3, respectively. The islet percentage and CD3 were calculated as islet- or CD3-positive area divided by total pancreas area of the section.

### Mathematical modeling to characterize in vitro cell kinetics and exposure-response and to predict in vivo GNTI-122 expansion in response to rapamycin.

The relationships between rapamycin concentrations, TCR stimulation, and GNTI-122 expansion and viability were characterized via a nonlinear mixed effect model trained on longitudinal in vitro GNTI-122 cell count and viability data (Supplemental Figure 6D). In vitro CISC activation was evaluated via E_max_ models to characterize the fraction of cells with active STAT5 signaling and the magnitude of p-STAT5 activation in cell culture (MFI) as a function of rapamycin concentrations. The relationship between GNTI-122 in vivo engraftment in the blood of NSG mice and simulated rapamycin exposures using the developed 2-compartment model was characterized for day 19 post–GNTI-122 injection via an E_max_ model.

### Translational model–based approach to guide clinical dosing of rapamycin in combination with GNTI-122 in T1D patients.

A literature search was performed to identify published human rapamycin PK data and models. Rapamycin PK profiles from these publications were digitized using WebPlotDigitizer (https://automeris.io/WebPlotDigitizer.html) and consolidated into a database (31–33, 49–60), which in turn was used to select and qualify published human PK rapamycin models. The predicted in vitro and in vivo response to rapamycin stimulation by the developed models was used to define a target trough concentration in humans. The selected PK rapamycin models were then used to identify an expected dose to achieve the target trough concentration with a daily rapamycin dosing regimen.

### Statistics.

GraphPad Prism version 9 was used to conduct all statistical analyses. For all studies, replicates were individual mice, cells, or donors. Sample means were assumed to be normally distributed. Specifics of the statistical tests used are indicated in each figure legend. No outliers were excluded.

### Study approval.

Healthy donor PBMCs were purchased from Stem Cell Technologies. T1D patient PBMCs were obtained from the Benaroya Research Institute (BRI) Registry and Repository and were approved by BRI’s Institutional Review Board (IRB 07109-588). Both healthy donors and T1D patient donors were HLA-DRB1*04:01. Donor demographics can be found in Table 1. All animal studies were conducted according to approved institutional animal care and use committee (IACUC) protocols at Charles River Laboratories (Durham, North Carolina, USA) and Hooke Laboratories (Lawrence, Massachusetts, USA).

### Data availability.

All underlying data can be found in the supplemental Supporting Data Values file.

## Author contributions

JHB, DJR, TFC, CP, KTM, and TJW conceived, designed, and provided oversight for the research. JYY, AL, PSL, HK, PZ, MSC, AC, MH, TD, RT, SJY, KC, and PJC executed experiments and prepared the figures. MR and GIU conceived and designed the experiments, provided oversight, and analyzed and interpreted the data. AL, HK, TD, and PJC set up the adoptive transfer T1D animal model and executed experiments. TG and EJ performed experiments on TCR specificity and analyzed results. PSL, DS, BG, MR, and KTM provided the tool, analyzed, and interpreted the rapamycin modeling data. KTM, TFC, MR, and GIU drafted the manuscript and revised it critically with input from all authors.

## Supplementary Material

Supplemental data

Supporting data values

## Figures and Tables

**Table 1:**
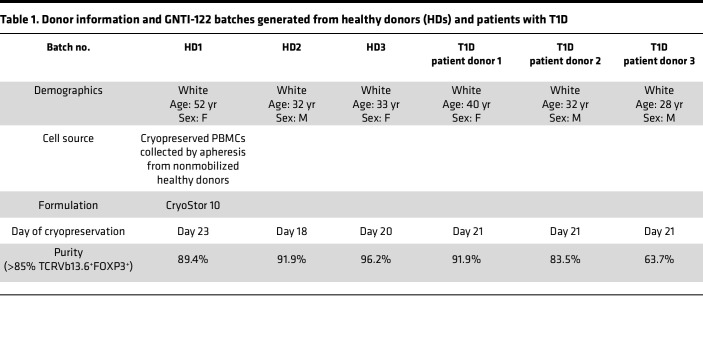
Donor information and GNTI-122 batches generated from healthy donors (HDs) and patients with T1D

## Version 1 03/22/2024

Electronic publication
