# Supplementary material for: GNTI-122: an autologous antigen-specific engineered Treg cell therapy for type 1 diabetes
Source: JCI Insight. 2024 Mar 22;9(6):e171844. doi: 10.1172/jci.insight.171844 (PMC11063937; doi:10.1172/jci.insight.171844)
Supplement: Supplemental data [file jciinsight-9-171844-s263.pdf]

Supplemental Material

Supplemental Methods

Generation of EngTregs used for stability studies

$CD4^+$ cells were isolated via magnetic enrichment from PBMCs. The cells were then genetically modified using CRISPR-Cas9 to target and knock-in at the *FOXP3* locus transgenes delivered by adeno-associated virus (AAV) vectors carrying the MND promoter driving the expression of chemically inducible signaling complex (CISC) and endogenous FOXP3. CISC receptor enables selective expansion and enrichment of engineered Tregs in the presence of rapamycin. The expanded cells were cryopreserved and used for further analyses and studies.

Isolation and expansion of human Tregs (cultured Tregs [cTregs])

For cTregs, $CD4^+$ $CD25^+$ $CD127^{lo}$ cells were isolated from PBMCs via flow cytometric cell sorting (BD Symphony by BD Biosciences). The cells were then activated using anti-CD3/CD28 Dynabeads (3:1; Thermo Fisher) for an initial expansion, before rest to match EngTreg protocol timelines. IL-2 (100 ng/mL) was supplemented to cTregs throughout and were stimulated again at Day 7 by CD3/CD28 beads (3:1). Expanded cells were cryopreserved and used for further analyses and studies described below.

RNA-Seq library preparation and sequencing

Cells were double-sorted by flow cytometry into TCL buffer (Qiagen) containing 1% 2-mercaptoethanol (Sigma) as previously described (1), and samples were processed by Azenta/Genewiz. Briefly, SMART-Seq v4 Ultra Low Input Kit for Sequencing was used for full-length cDNA synthesis and amplification (Clontech), and Illumina Nextera XT library was used for sequencing library preparation. Briefly, cDNA was fragmented, and adaptor was added using transposase, followed by limited-cycle PCR to enrich and add index to the cDNA fragments. The final library was assessed with Agilent TapeStation.

The sequencing libraries were multiplexed and clustered on lane(s) of a flowcell. After clustering, the flowcell was loaded on the Illumina HiSeq instrument according to manufacturer's instructions. The samples were sequenced using a 2 × 150 paired-end configuration. Image analysis and base calling were conducted by the HiSeq Control Software. Raw sequence data (bcl files) generated from Illumina HiSeq were converted into fastq files and de-multiplexed using Illumina's bcl2fastq 2.17 software. One mismatch was allowed for index sequence identification.

Following library preparation, reads were aligned using STAR (2). The gene-level quantification was calculated by Salmon (3) feature counts (http://subread.sourceforge.net/) and raw read counts tables were normalized by median of ratios method with DESeq2 package from Bioconductor and converted to Gene Pattern gct and cls format. Poor quality samples with less than 3 million uniquely mapped reads were excluded from normalization and normalized read counts were filtered for expression (>10) to avoid background confounders. After normalization, reads were further filtered by minimal expression in Multiplot Studio (GenePattern; Broad Institute). All signature gene sets are referenced in the corresponding figure legends. Heat maps were generated with Morpheus (https://software.broadinstitute.org/morpheus), typically using log2-transformed FoldChange relative to

mean of controls. The CoreTreg gene signature (4) used for analysis included FOXP3, IL2RA, IL2RB, IKZF2, TNFRSF1B.

Proinflammatory challenge culture conditions

2×10^5 EngTregs and cTregs were cultured in 96-well plates in RPMI + 20% FBS with or without αCD3/CD28 Dynabeads (1:1 ratio), IL-2 (50 ng/mL), a cocktail of IL-6 (50 ng/mL), TNFα (50 ng/mL), and IL-12 (10 ng/mL) with or without rapamycin (10 nM) for 72 hours prior to flow cytometric analysis.

Pathogen-derived peptide sequences

Pathogen-derived peptide sequences were selected using a multistep screening approach. First, the islet-specific glucose-6-phosphatase catalytic subunit-related protein (IGRP) (305-324) minimal epitope 'FLQIPTHEE' was queried against peptides from pathogenic species with known risks to humans by protein BLAST using the non-redundant protein sequence database, yielding 298 pathogenic sequences with ≤3 amino acid mismatches (https://blast.ncbi.nlm.nih.gov/Blast.cgi?PROGRAM=blastp&PAGE_TYPE=BlastSearch&LINK_LOC=blasthome, accessed 08/2021). These homologous pathogenic peptides were then assigned a relative binding score (RBA) to DRB1*04:01 using a previously published algorithm (5). All peptides with a predicted RBA above 0.0498, one-tenth the IGRP minimal epitope binding score (RBA = 0.498), were selected as potential DRB1*04:01 binders. In total, 67 unique peptides met this cutoff and were then evaluated to assess the similarity of their T-cell receptor (TCR) contact residues to the IGRP 305-324 peptide. Based on crystal structures of peptide/HLA-DR and peptide/HLA-DR/TCR complexes, residues at positions p1, p2, p3, p5, p8, and p10 that flank or are within the minimal epitope HFLQIPTHEEH were considered potential TCR contact residues (6). The BLOSUM-62 matrix was then used to evaluate the similarity of the residues at the potential TCR contact sites in the selected peptides compared to the corresponding residues in HFLQIPTHEEH. The default BLOSUM-62 matrix was modified based on the tolerated residue substitutions observed in the alanine scanning experiment to generate a custom TCR recognition matrix for each potential contact residue and combined these values to calculate a TCR recognition score for each of the 67 peptides. Finally, to produce a refined list of potentially cross-reactive sequences, an aggregate score was generated by multiplying the DRB1*0401 binding score and TCR recognition score. Peptides with a score above the median aggregate score of 3.6 were chosen for further analysis, resulting in 13 predicted epitopes from human pathogens that were tested for cross-recognition using a CD137 upregulation assay (see results in **Figure 4B**).

TCR selection

TCR selection was performed using previously published methods (5). Briefly, TCRs derived from clonally expanded $CD4^+$ cells were isolated from patients with T1D. Candidate TCRs were engineered into Tregs, referred to as EngTregs. These EngTreg candidates expressing a human TCR were then evaluated using a panel of assays to select a final TCR for inclusion in the GNTI-122 cells. These assays include direct and bystander suppression and avidity.

Cell characterization and phenotypic profiling

Cells were first stained with LIVE/DEAD Fixable Aqua (Invitrogen) for 30 minutes at 4 °C and then stained for 20 minutes at 4 °C with the corresponding surface antibodies. The cells were then washed, fixed, and

permeabilized (eBioscience™ Foxp3 / Transcription Factor Staining Buffer Set) for 45 minutes at room temperature before proceeding with the intracellular staining for 1 hour at room temperature. After the incubation, the cells were washed and resuspended in PBS for acquisition.

GNTI-122 engraftment

Eight- to 10-week-old female NSG mice were randomized into treatment groups based on body weight. Starting on Day 1, rapamycin was administered intraperitoneally once every other day for the duration of 17 days. On Day 3, the mice received whole-body irradiation (200 cGy) to promote the initial engraftment of GNTI-122 in a mouse host, followed by GNTI-122 administration 2 hours later. Half of the animals per group were euthanized on Day 19, 48 hours after the last rapamycin dose, and the second half on Day 31, 14 days after completion of rapamycin treatment. Persistence of GNTI-122 was assessed by flow cytometry in blood, spleen, bone marrow, and liver (**Supplemental Figure 6A**).

Supplemental Figures

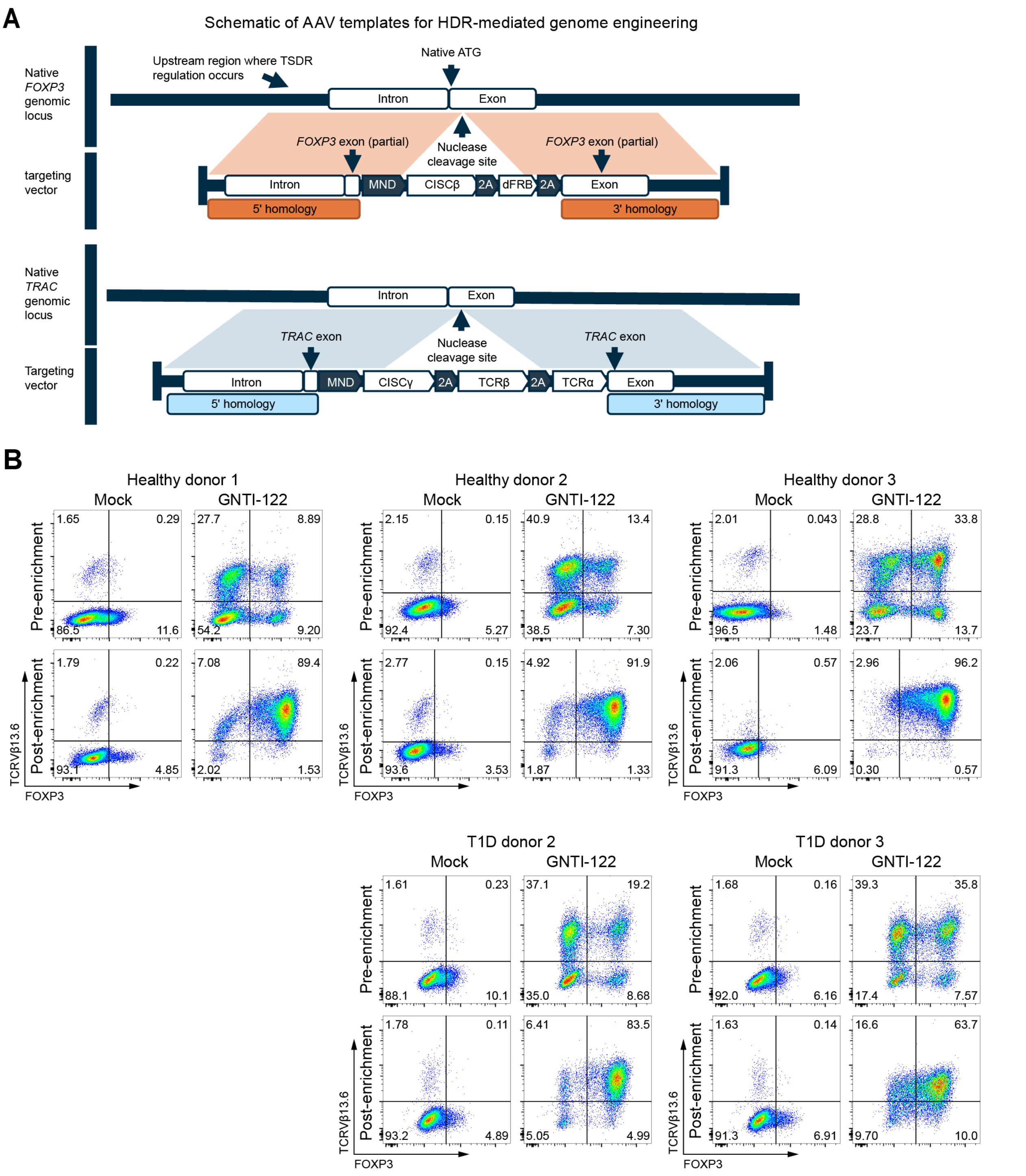

Supplemental Figure 1. (A) Schematic of AAV templates and associated CRISPR/Cas9 target cleavage site for HDR-mediated targeted integration of transgenes for GNTI-122. **(B)** FACS analysis of GNTI-122 and Mock cells generated from healthy donors (HDs) and patients with type 1 diabetes (T1D) (T1D Donor 1 in Figure 1D). AAV, adeno-associated virus; HDR, homology-directed repair; TSDR, Treg-specific demethylated region; ATG, start codon.

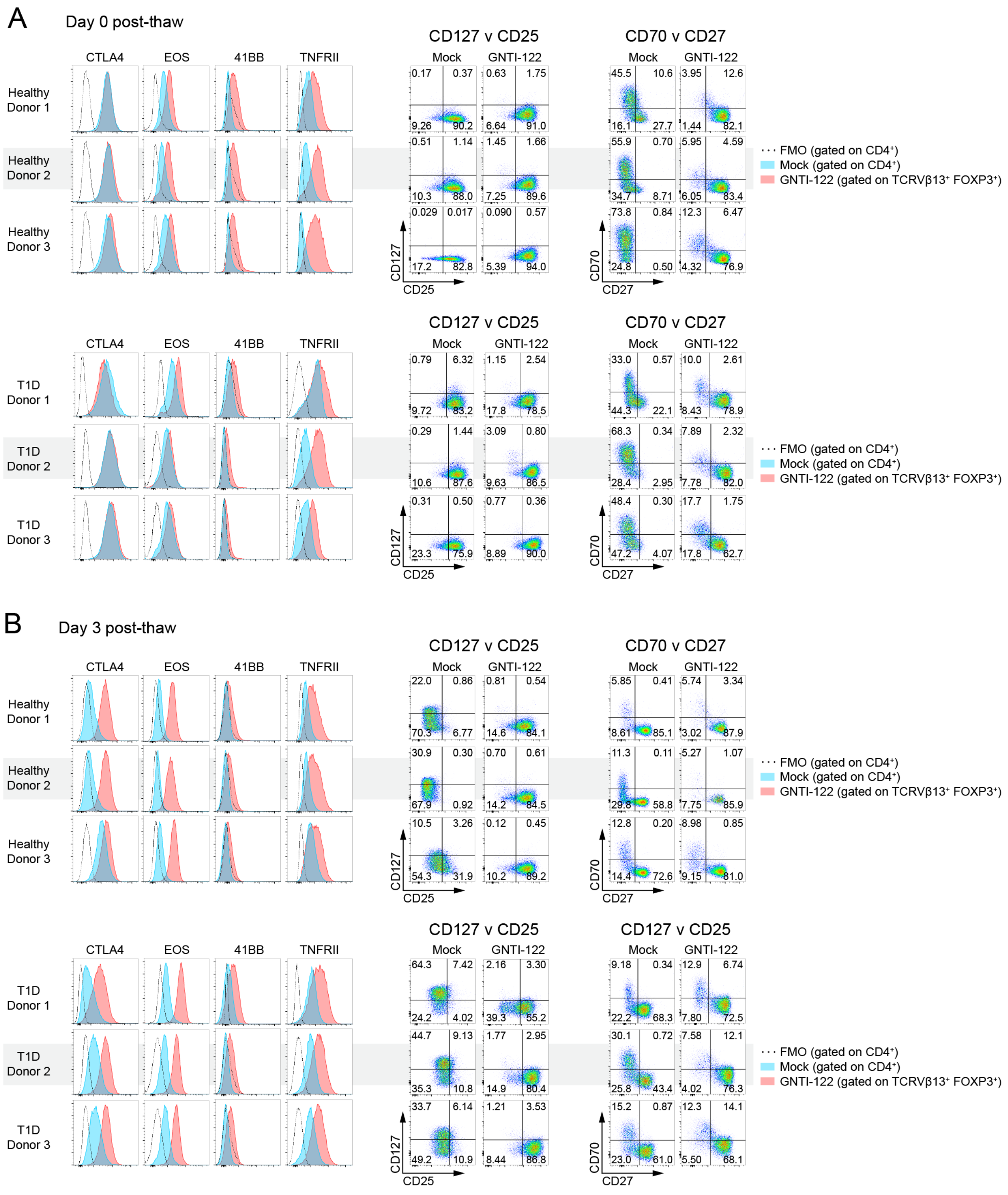

Supplemental Figure 2. Flow cytometry plots of all healthy donor (HD) and type 1 diabetes (T1D) patient doner–derived mock engineered (Mock) and GNTI-122, immediately after thaw and after 3 days of rest in low IL-2 culture. FMO, fluorescence minus one

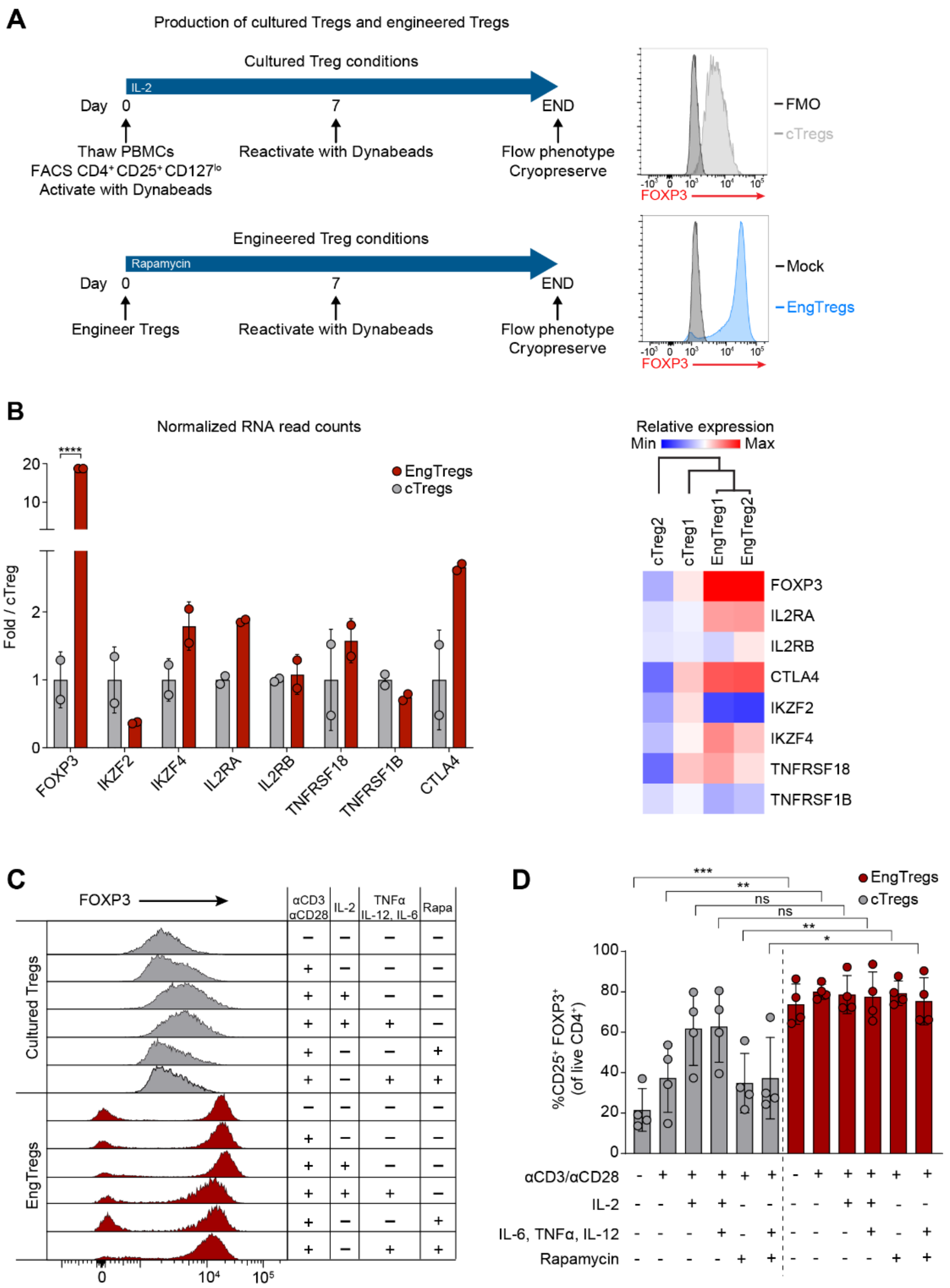

Supplemental Figure 3. **(A)** Schematic diagram of cultured Treg (cTreg) and engineered Treg (EngTreg) production process with representative flow plots of the resulting cells' FOXP3 expression levels. **(B)** Graph and heat map of gene expression comparison between donor matched EngTregs and cTregs generated from 2 independent donors reveal that typical Treg transcripts such as CTLA4, IKZF4 (EOS), IL2RA, and TNFRSF18 are not significantly different, while FOXP3 transcript is significantly higher (n=2, mean ± SEM). **(C)** Representative flow of FOXP3 expression after culture and **(D)** cumulative data of 4 independent experimental results showing maintained FOXP3+ CD25+ phenotype after 72 hours without IL-2 and/or anti-CD3/CD28 stimulation (n=4, mean ± SEM). Paired *t* test; **P* < .05, ***P* < .01, ****P* < .001, *****P* < .0001. ns, not significant; FMO, fluorescence minus one; Mock, mock-engineered cells; rapa, rapamycin.

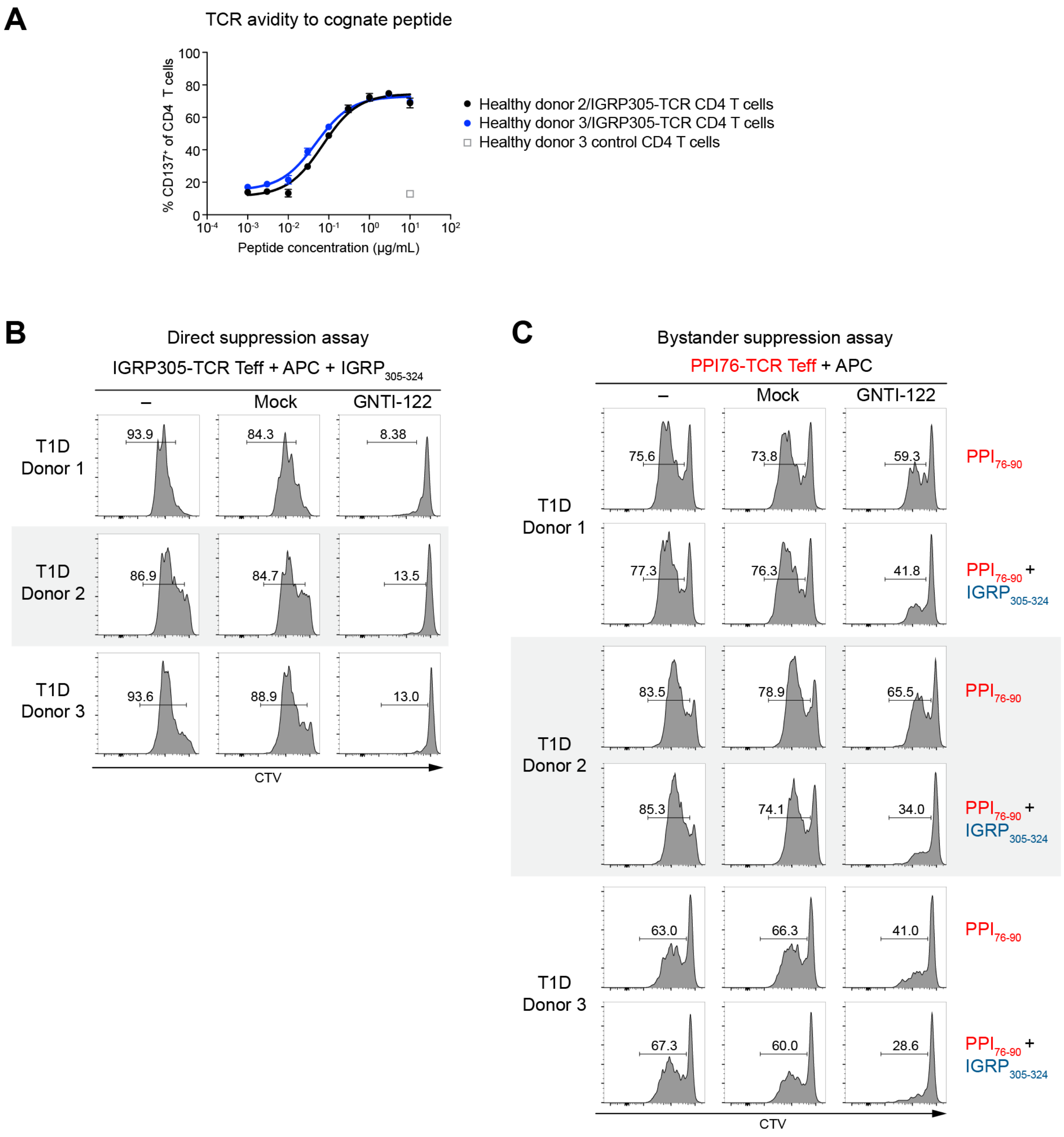

Supplemental Figure 4. (A) Sensitivity of IGRP305 T-cell receptor (TCR) for its cognate peptide in the assay system. K562 target cells expressing HLA-DR4 (K562/DR4) were pulsed with 0.001 to 10 µg/mL of the $IGRP_{305\text{-}324}$ peptide and cocultured with IGRP305-TCR transduced primary human CD4 T cells from 2 donors at 3:1 effector:target ratio. CD137 expression was measured after 20 hours. Near-maximal response is achieved with 1 µg/mL of peptide in this system. (mean ± SEM). **(B)** Individual flow cytometry plots of direct suppression assay. **(C)** Individual flow cytometry plots of the bystander suppression assay. Teff, T effector cells; APC, antigen-presenting cells; IGRP, islet-specific glucose-6-phosphatase catalytic subunit–related protein; Mock, mock-engineered cells; CTV, cell trace violet; PPI, preproinsulin

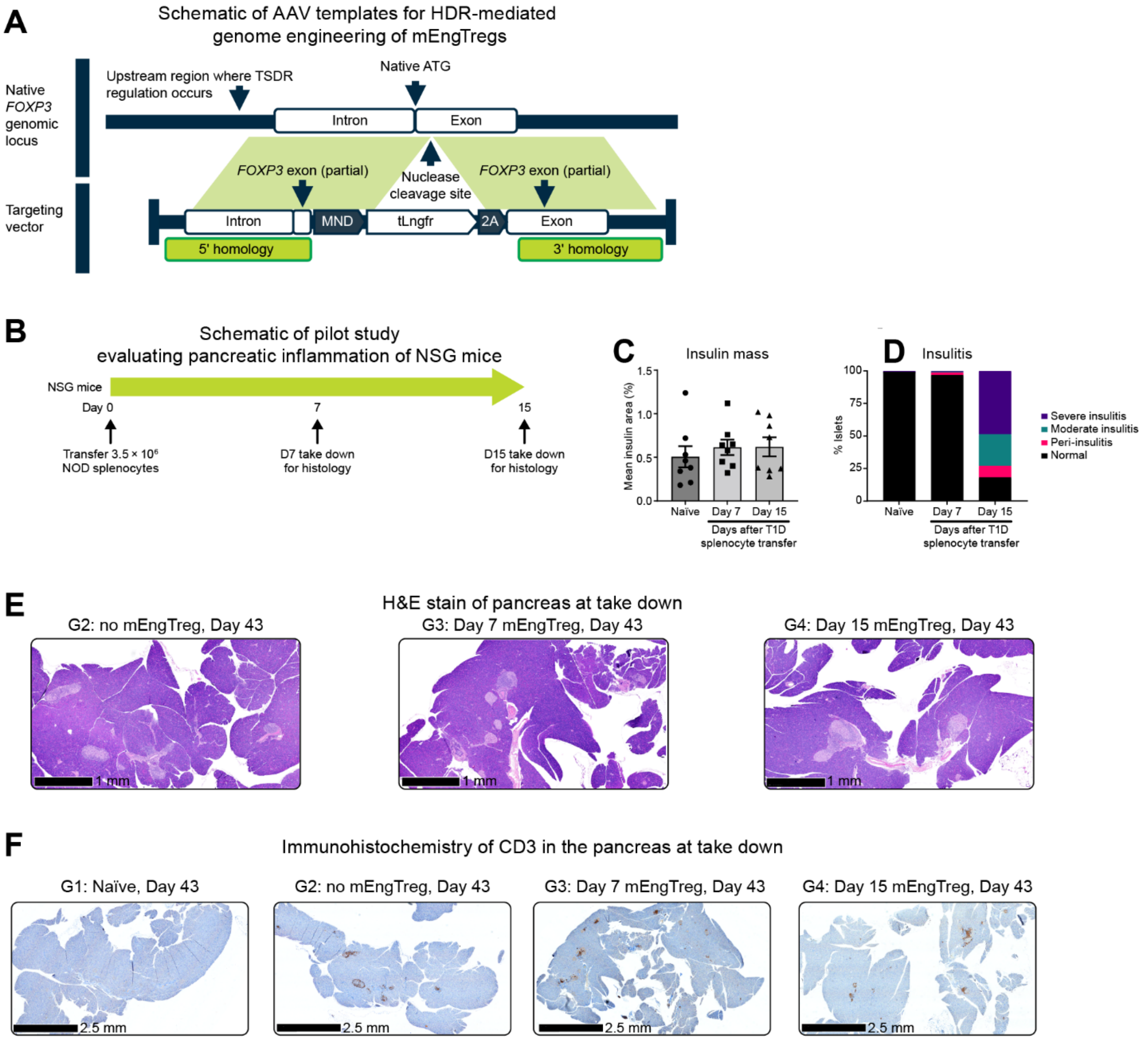

Supplemental Figure 5. (**A**) Schematic of AAV template and associated CRISPR/Cas9 target cleavage site for HDR-mediated targeted integration of the mEngTreg transgene. (**B**) Schematic diagram of the model development. (**C**) Quantification of beta cell mass by insulin staining of pancreata. Approximately 20 pancreatic islets quantified per mouse (n=8, mean ± SEM). (**D**) Severity scores of pancreatic islet inflammation quantified via H&E staining. Representative (**E**) H&E-stained histology and (**F**) immunohistochemistry stain of CD3 of pancreas at take down (D43) for the study represented in **Figure 5G**. AAV, adeno-associated virus; HDR, homology-directed repair; mEngTregs, mouse Engineered Tregs; TSDR, Treg-specific demethylated region; ATG, start codon; tLngfr, truncated low-affinity nerve growth factor receptor.

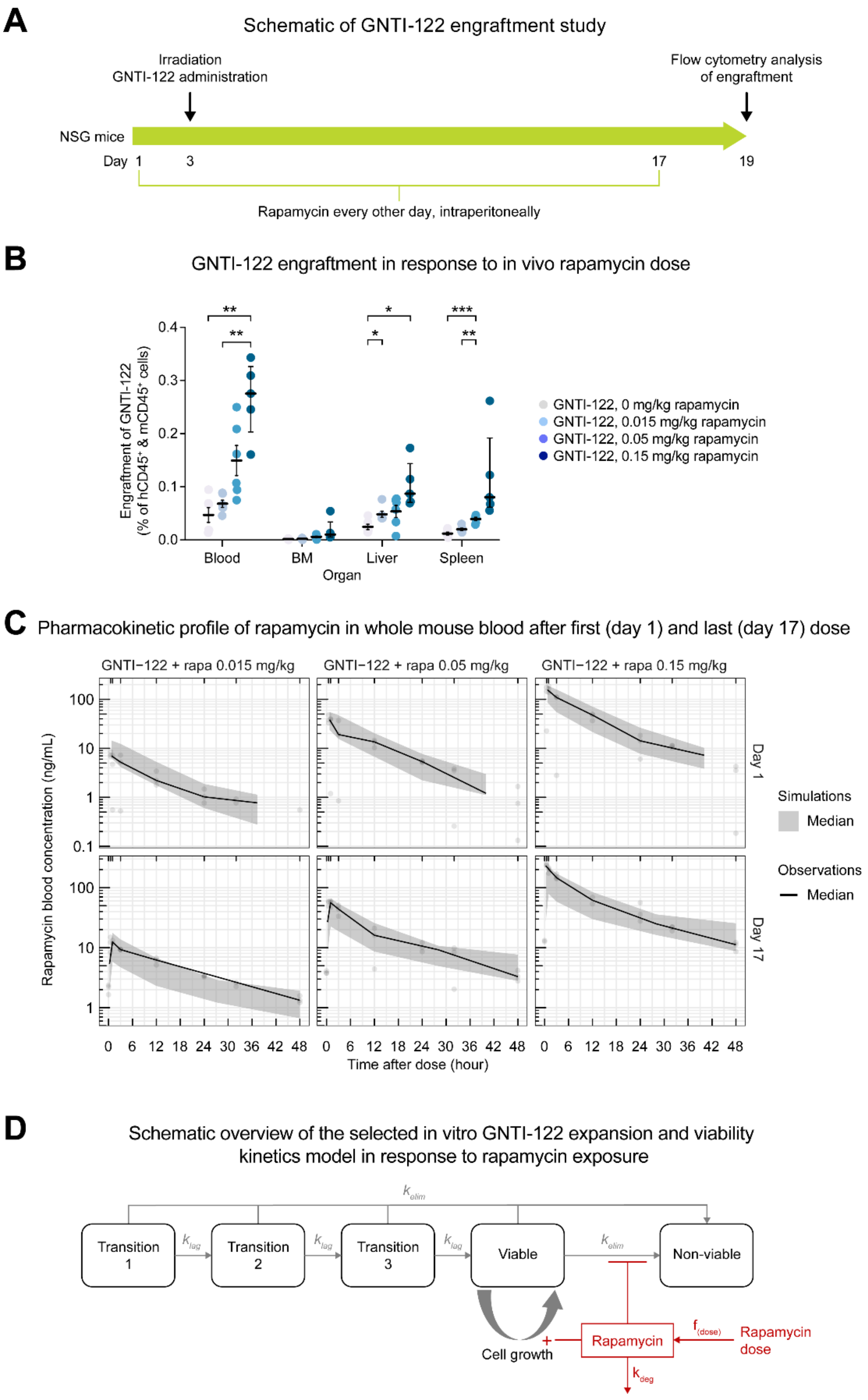

Supplemental Figure 6. (**A**), Schematic diagram of in vivo study assessing engraftment of GNTI-122 in NSG mice supported by rapamycin treatment. (B) Rapamycin administered every other day promoted GNTI-122 engraftment in a dose-dependent manner in blood, bone marrow, liver and spleen (n=5, mean

± SEM) 1-way ANOVA, $*P < .05$, $**P < .01$, $***P < .001$, $****P < .0001$. (**C**) The corresponding pharmacokinetic profiles of rapamycin in whole mouse blood after the first and last dose. Median measured values (line) and modelling based on 1000 simulations (gray area; median and 95% confidence interval). (**D**) Schematic overview of the selected model to describe in vitro cellular kinetics of GNTI-122 expansion and viability in response to rapamycin exposure. The model considers two GNTI-122 model species, i.e., viable (V) and non-viable (NV) cells. Viable cells undergo exponential expansion within a predefined time window in rapamycin-dependent manner in presence of TCR stimulation. The expansion process is assumed to take place after a delay at the start of the experiment, which is implemented via three transition compartments. Viable cells further transition towards nonviable cells; this process is inhibited in presence of rapamycin, independently of TCR stimulation. The rapamycin concentrations in the selected model are not constant but change over time since rapamycin in the media is further eliminated or metabolized following a first-order process with the rate constant kdeg. hCD45, human CD45; mCD45, mouse CD45; BM, bone marrow; rapa, rapamycin; K_{elim}, elimination constant; K_{lag}, transition rate constant of the cellular expansion process; F(dose), rapamycin bioavailability; K_{deg}, degradation constant.

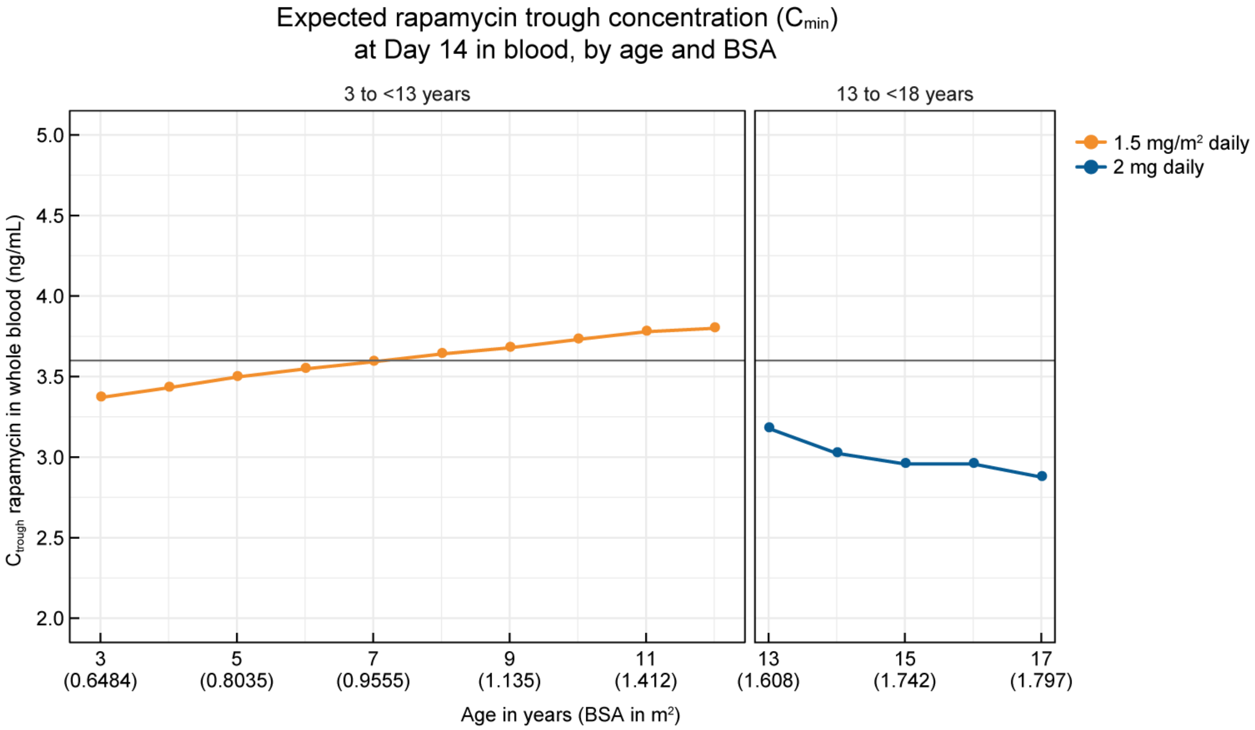

Supplemental Figure 7. A model showing the changes in predicted pharmacokinetic profile of rapamycin with age in children and adolescents. C_{min} (minimal concentration of rapamycin before administration of the next daily dose) is shown on y-axis. Age and corresponding body surface area are shown on x-axis.

Supplemental Tables

Supplemental Table 1. Cell surface markers

**Marker**	**Role/Function**
TCRVb13.6	IGRP305-TCR-specific TCRVβ domain
CD3	Pan T-cell marker; expressed on Tregs
CD4	T-cell subset marker; expressed on Tregs
CD25	IL-2 receptor alpha subunit; constitutively expressed on Tregs
CD127	Teff marker; downregulated on Tregs
ICOS (CD278)	Expressed on Tregs
CD39	Ectonucleotidase that degrades ATP to AMP; expressed on Tregs
CD73	Ectonucleotidase that converts AMP to adenosine; expressed on Tregs
FOXP3	Transcription factor highly expressed in Tregs
CTLA-4 (CD152)	CD80/86 agonist blocks binding to CD28 and DC's ability to activate Teffs
EOS	Transcription factor stably expressed on Tregs
CD70	Exhaustion marker of Tregs
CD27	TNF receptor superfamily that binds to CD70; expressed on Tregs
GITR (CD357)	TNF receptor superfamily; expressed on Tregs
TNFRII (CD120b)	TNF receptor associated with activation and stability of Tregs
4-1BB (CD137)	TNF receptor superfamily; expressed on activated Teffs and Tregs
TIGIT (VSTM3)	Co-inhibitory molecule expressed on a subset of Tregs
PD-1 (CD279)	Expressed on activated T cells and negatively regulates activation
LAG-3	Ig superfamily expressed on activated T cells and Tregs

Supplemental Table 2. Alanine mutant peptide sequences

IGRP$_{305-324}$	QLYH**FLQIPTHEE**HLFYVLS
P1	QLYAFLQIPTHEEHLFYVLS
P2	QLYHALQIPTHEEHLFYVLS
P3	QLYHFAQIPTHEEHLFYVLS
P4	QLYHFLAIPTHEEHLFYVLS
P5	QLYHFLQAPTHEEHLFYVLS
P6	QLYHFLQIATHEEHLFYVLS
P7	QLYHFLQIPAHEEHLFYVLS
P8	QLYHFLQIPTAEEHLFYVLS
P9	QLYHFLQIPTHAEHLFYVLS
P10	QLYHFLQIPTHEAHLFYVLS
P11	QLYHFLQIPTHEEALFYVLS

Supplemental Table 3. Pathogenic species with known risks to humans

Virus	Bacteria	Fungal	Parasites
HSV-1 and -2; Herpes simplex virus-1 and -2	Mycobacteria<br>- tuberculosis<br>-avium complex<br>others	Aspergillosis	*Cryptosporidium*
CMV; Cytomegalovirus	*Nocardia*	Histoplasmosis	Microsporida
VZV; varicella-zoster virus	*Listeria*	Coccidioidomycosis	Toxoplasmosis
EBV; Epstein-Barr virus	*Salmonella*	Blastomycosis	Strongyloidiasis
HHV6; human herpes virus 6	*Bartonella*	Mucormycosis	
HHV8; human herpes virus 6	*Neisseria gonorrhoeae*	*Candida*	
HBV; hepatitis B virus	*Streptococcus pneumoniae*	*Pneumocystis jiroveci*	
HCV; hepatitis C virus	*Legionella pneumophila*		
BK; polyomavirus hominus 1	*Mycoplasma pneumoniae*		
Adenovirus	*Staphylococcus aureus*		
Molluscum	*Staphylococcus epidermidis*		
HPV; human papilloma virus	*Treponema pallidum* (syphilis)		
JC; polyomavirus (PML) John Cunningham virus	*Chlamydia trachomatis*		
SARS-CoV-2; severe acute respiratory syndrome coronavirus 2			
Influenza A and B (most recent strains)			
HMPV; human metapneumovirus			
RSV; respiratory syncytial virus			
HPIV; human parainfluenza virus			

Supplemental Table 4. Putative $IGRP_{305\text{-}324}$ cross-reactive peptides

Label	Description	Scientific Name	15 mer Sequence	Risk to humans?
Pathogen 2	TPA: phosphopentomutase	*Legionella* sp.	SDSVLQIAAHEEHFG	Yes
Pathogen 5	DUF4435 domain-containing protein	*Bacillus toyonensis*	YDEVLQIPTHQENTQ	Yes
Pathogen 9	UDP-N-acetylglucosamine--N-acetylmuramyl-(pentapeptide) pyrophosphoryl-undecaprenol N-acetylglucosamine transferase		AASFLHIPYFTHECD	
Pathogen 10	TPA: UDP-N-acetylglucosamine-N-acetylmuramyl-(pentapeptide) pyrophosphoryl-undecaprenol N-acetylglucosamine transferase		AARFLHIPYYTHECD	
Pathogen 11	undecaprenyldiphospho-muramoylpentapeptide beta-N-acetylglucosaminyltransferase	*Treponema* sp.	AAKFLHIPVFTHECD	Yes
Pathogen 12	exodeoxyribonuclease V subunit beta	*Chlamydia caviae*	CSPFLQIPSYEPIEY	Yes
Pathogen 15	hypothetical protein V495_01361	*Pseudogymnoascus* sp. VKM F-4514 (FW-929)	SAMFLDIPTHPEEHS	Yes
Pathogen 16	hypothetical protein GX51_07420	*Blastomyces parvus*	KYEFLPIPTYEEATS	Yes
Pathogen 17	hypothetical protein T551_03071	Pneumocystis jiroveci RU7	FTDFLNIPSYEELND	Yes
Pathogen 18	hypothetical protein KMI_05g09430	*Encephalitozoon hellem*	LSEFLKIPNHEITLL	Yes
Pathogen 19	hypothetical protein ECU05_1220	*Encephalitozoon cuniculi* GB-M1	QQKFLKNIDTHEEAE	Yes
Pathogen 20	WD domain, G-beta repeat-containing protein	*Toxoplasma gondii* ARI	ESLFLQLSTHTEASA	Yes
Pathogen 23	ATP-dependent DNA helicase	*Legionella lansingensis*	DQLFLALPAHEERIS	Yes

Supplemental Table 5. Healthy donor digital PCR primer probe sequences

Detection Primer/Probe	Sequence
FOXP3 F Primer	CGGCGACGTGGAAGAGAATC
FOXP3-KI R Primer	GGCTGTGGTTCAGCCTGACT
FOXP3 FAM Probe	AGGCTCTCCCCGACCTCCC
TRAC F Primer	TCTGTGCAGCAACCCGT
TRAC-KI R Primer	AGGGTTTTGGTGGCAATGGA
TRAC FAM Probe	CCTTCTTCCCCAGCCCAGGT
GAPDH F Primer	TCATGCCTTCTTGCCTCTTGT
GAPDH R Primer	TTGGATGAGAAAGGTGGGAGC
GAPDH HEX Probe	CGTCGTGGAGTCCACTGG

Supplemental Table 6. T1D patient donor digital PCR primer probe sequences

Detection Primer/Probe	Sequence
FOXP3 F Primer	CACTTCTCGCCTTCTCCACT
FOXP3-KI R Primer	CTGCTGTTCCAACTGTTCTTGG
FOXP3 HEX Probe	CAGCATCATCACTTGCCAGGACT
TRAC F Primer	GCTTAGACGCAGGTGTTCTGA
TRAC-KI R Primer	CTGCTGTTCCAACTGTTCTTGG
TRAC FAM Probe	CTGGCAAGTCACGGTCTCATGCT
RPPH1 F Primer	CTGGCCCTAGTCTCAGACCTT
RPPH1 R Primer	GCGGAGGGAAGCTCATCAG
RPPH1 FAM/HEX Probe	CCACGAGCTGAGTGCGTCCTGT

Supplemental Table 7. Human antibody list

Marker	Clone	Fluorophore	Marker localization	Manufacturer	Cat No.	Dilution
P2A	3HA	AF488	Intracellular	Novus Biologicals	NBP2-59627AF488	1:100
TCRVb13.6	REA554	PE	Surface	Miltenyi Biotec	130-126-523	1:50
CD3	UCHT1	APC-Cy7	Surface	BioLegend	300426	1:200
CD4	RPA-T4	AF700	Surface	BioLegend	300526	1:500
CD4	OKT4	BV711	Surface	BioLegend	317440	1:200
CD25	2A3	BV605	Surface	BD Biosciences	562660	1:100
CD25	BC96	APC	Surface	BioLegend	302610	1:100
CD127	A019D5	PeCy7	Surface	BioLegend	351320	1:200
CD127	A019D5	BV605	Surface	BioLegend	351334	1:100
ICOS (CD278)	C398.4A	BV421	Surface	BioLegend	313524	1:100
ICOS (CD278)	C398.4A	PerCP Cy5.5	Surface	BioLegend	313518	1:200
CD39	TU66	BV711	Surface	BD Biosciences	563680	1:200
CD73	AD2	BV785	Surface	BioLegend	344028	1:100
FOXP3	259D	AF488	Intracellular	BioLegend	320212	1:100
FOXP3	PCH101	PeCy7	Intracellular	Invitrogen	25-4776-4	1:100
CTLA-4 (CD152)	L3D10	APC	Intracellular	BioLegend	349908	1:200
CTLA-4 (CD152)	BNI3	BV785	Intracellular	BioLegend	369624	1:50
EOS	W16032 A	APC	Intracellular	BioLegend	399306	1:100
CD70	113-16	PeCy7	Surface	BioLegend	355112	1:100
CD27	O323	BV650	Surface	BioLegend	302828	1:50
GITR (CD357)	108-17	BV605	Surface	BioLegend	371214	1:200
TNFRII (CD120b)	3G7A02	APC	Surface	BioLegend	358406	1:100
4-1BB	4B4-1	PeCy7	Surface	BioLegend	309818	1:100

Marker	Clone	Fluorophore	Marker localization	Manufacturer	Cat No.	Dilution
TIGIT (VSTM3)	A15153 G	BV421	Surface	BioLegend	372709	1:50
PD-1	EH12.2H 7	BV650	Surface	BioLegend	329950	1:100
LAG-3	11C3C6 5	BV785	Intracellular	BioLegend	369322	1:100
IL-2	MQ1-17H12	PeCy7	Intracellular	BioLegend	500326	1:25
IFNg	4S.B3	AF647	Intracellular	BioLegend	502516	1:25
LAP	S20006A	PeCy7	Surface	BioLegend	300007	1:20
GARP	7B11	BV421	Surface	BD Biosciences	563956	1:50

Supplemental Table 8. Mouse antibody list

Marker	Clone	Fluorophore	Marker localization	Manufacturer	Cat No.	Dilution
Lngfr	REA648	PE	Surface	Miltenyi Biotec	130-118-793	1:100
CD4	GK1.5	BV605	Surface	BioLegend	100451	1:200
Foxp3	FJK-16S	PerCP-Cy5.5	Intracellular	Invitrogen	45-5773-82	1:50
Helios	22F6	PE-Cy7	Surface	BioLegend	137235	1:50
CD45	30-F11	FITC	Surface	BioLegend	103121	1:200
CD8a	53-6.7	BV650	Surface	BioLegend	100741	1:200
CD62L	MEL-14	SB780	Surface	Invitrogen	78-0621-82	1:200
CD44	IM7	AF780	Surface	Invitrogen	25-0441-82	1:200
CD3	17A2	Pacific Blue	Surface	Invitrogen	14-0032-82	1:200
vB4	REA729	APC	Surface	Miltenyi Biotec	130-110-982	1:200
LIVE/DEAD	N/A	Invitrogen Fixable Aqua	Intracellular	Invitrogen	L34966	1:1000

Supplemental References

1. Zarin P, et al. Treg cells require Izumo1R to regulate gammadeltaT cell-driven inflammation in the skin. *Proc Natl Acad Sci U S A.* 2023;120(14):e2221255120.
2. Dobin A, et al. STAR: ultrafast universal RNA-seq aligner. *Bioinformatics.* 2013;29(1):15–21.
3. Patro R, et al. Salmon provides fast and bias-aware quantification of transcript expression. *Nat Methods.* 2017;14(4):417–419.
4. Zemmour D, et al. Single-cell gene expression reveals a landscape of regulatory T cell phenotypes shaped by the TCR. *Nat Immunol.* 2018;19(3):291–301.
5. Yang SJ, et al. Pancreatic islet-specific engineered Tregs exhibit robust antigen-specific and bystander immune suppression in type 1 diabetes models. *Sci Transl Med.* 2022;14(665):eabn1716.
6. Rossjohn J, et al. T cell antigen receptor recognition of antigen-presenting molecules. *Annu Rev Immunol.* 2015;33:169-200.
